# Genetic metrics decode *Plasmodium falciparum* diversity: complexity of infection, parasite connectivity, and transmission intensity in Mainland Tanzania’s diverse regions

**DOI:** 10.3389/fgene.2026.1695073

**Published:** 2026-04-29

**Authors:** Dativa Pereus, Abebe A. Fola, Catherine Bakari, Misago D. Seth, Rebecca DeFeo, Beatus M. Lyimo, Celine I. Mandara, Rashid A. Madebe, Doris Mbata, Zachary R. Popkin-Hall, Ramadhan Moshi, Ruth B. Mbwambo, Daniel Mbwambo, Sijenunu Aaron, Abdallah Lusasi, Samwel Lazaro, David J. Giesbrecht, Benard Kulohoma, Jonathan J. Juliano, Jeffrey A. Bailey, Gerald Juma, Victor A. Mobegi, Deus S. Ishengoma

**Affiliations:** 1 National Institute for Medical Research, Dar es Salaam, Tanzania; 2 Department of Biochemistry, University of Nairobi, Nairobi, Kenya; 3 Departmet of Medical Botany, Plant Breeding and Agronomy, Muhimbili University of Health and Allied Sciences, Dar es Salaam, Tanzania; 4 Department of Biomedical Research and Clinical trial, Ifakara Health Institute, Dar es Salaam, Tanzania; 5 Department of Pathology and Laboratory Medicine, Brown University, Providence, RI, United States; 6 School of Life Sciences and Bioengineering, Nelson Mandela African Institute of Science and Technology, Arusha, Tanzania; 7 Division of Infectious Diseases, University of North Carolina School of Medicine, University of North Carolina, Chapel Hill, NC, United States; 8 National Malaria Control Programme, Dodoma, Tanzania; 9 The Connecticut Agricultural Experiment Station, New Haven, CT, United States; 10 International Aids Vaccine Initiative (IAVI), Nairobi, Kenya; 11 Ortholog, Nairobi, Kenya

**Keywords:** genetic diversity, genetic metrics, Mainland Tanzania, malaria molecular surveillance, *Plasmodium falciparum*, symptomatic patients, transmission strata

## Abstract

**Introduction:**

Recent evidence indicates that an increasing number of endemic countries have deployed and are using genomic surveillance to determine and monitor the trends and patterns of malaria transmission. This study aimed to evaluate and identify the most informative genetic metrics for establishing and monitoring the genetic diversity of *Plasmodium falciparum* and its correlation with malaria transmission intensities in Mainland Tanzania.

**Methods:**

A cross-sectional survey of symptomatic patients was conducted in 100 health facilities from February to July 2021 and covered 10 regions categorized into four strata based on transmission intensity. Parasite samples (n = 12,875) were collected as dried blood spots, and all samples with *P. falciparum* positive test by rapid diagnostic tests (n = 7,199) were sequenced using molecular inversion probes. We targeted 1,832 single nucleotide polymorphisms distributed across the 14 *P. falciparum* chromosomes. Raw sequence data were analyzed using MIPTools and the final dataset was used to estimate different genetic metrics.

**Results:**

The countrywide mean complexity of infection (COI) was 1.5, with 1,878 (59.6%) of parasite samples being monoclonal. The mean COI was significantly higher in high and moderate transmission strata (p < 0.001) compared to low and very low transmission strata. The odds of polyclonal infections were significantly lower in moderate, low, and very low strata compared to the high transmission stratum (p < 0.001). Parasite genetic differentiation among regions was very low, with fixation index (*F*_*ST*_) values of 0–0.006. Countrywide parasite populations indicated weak genetic relatedness with pairwise identity by descent (IBD < 0.1). Few pairs (1.8%) met the thresholds of IBD ≥ 50%, and among these, the average pairs of parasites sharing ≥50% IBD were 0.89 and 0.98 for those sharing ≥90%. Discriminant analysis of principal components (DAPC) revealed overlapping parasite population clusters, suggesting genetic similarity among them.

**Discussion:**

The study revealed high complexity and polyclonality, particularly in regions with high transmission intensities. The significant association between COI, polyclonality, and transmission intensity suggests these metrics can be integrated within the current malaria surveillance system and may be useful in assessing trends and patterns of malaria transmission. Further validation is needed to link these measures with the current control strategies and evaluate their use in determining the impact of different malaria interventions in Mainland Tanzania.

## Introduction

Genomic surveillance, which combines epidemiological applications with genomics and bioinformatics methods, is increasingly becoming more recognized as a powerful tool for infectious disease surveillance, including malaria (Watson et al., 2021; Mensah et al., 2022). Malaria molecular surveillance (MMS), which has been endorsed by the World Health Organization (WHO) (WHO Science Council, 2022), is now being deployed in various endemic countries to complement traditional malaria surveillance efforts (Mensah et al., 2022; Neafsey et al., 2021). MMS using parasite genomic data, epidemiological data and patient metadata complement traditional methods by detecting low density infections, monitor emerging drug resistance markers, resolving local and regional transmission networks, measuring the impact of interventions on parasite genomic diversity, and identifying sources and sinks of infections (Watson et al., 2021; Morgan et al., 2020; Gwarinda et al., 2023; Holzschuh et al., 2023; Connelly et al., 2023). It is also used to detect signatures of adaptation and natural selection (Morgan et al., 2020; Lyimo et al., 2024); identify imported cases (Morgan et al., 2020; Connelly et al., 2023), loci associated with vaccine escape (Lyimo et al., 2024; Kisambale et al., 2025), and antimalarial drugs (Moser et al., 2020; Juliano et al., 2024; Ishengoma et al., 2024a) or diagnostic resistance (Rogier et al., 2024). Thus, MMS in combination with epidemiological data has proved to be an essential tool for assessing programme performance, the impact of current interventions, and determining if there is a need for intervention adjustments as well as resource allocation for the targeted population (Watson et al., 2021; Mensah et al., 2022; Ishengoma et al., 2019).

Despite intensified interventions over the past two decades, the burden of malaria, mainly caused by *Plasmodium falciparum,* remains high in Sub-Saharan Africa (World Health Organization, 2024). In 2023, Tanzania ranked fourth in malaria deaths, accounting for 4.3% of global malaria fatalities (World Health Organization, 2024). Recent interventions have created varied transmission patterns in Mainland Tanzania, with some areas experiencing persistently high malaria burden while other parts of the country (40%) experience low to very low transmission intensities and disease burden (Moser et al., 2020; Thawer et al., 2020; National malaria control program, 2020).

The country is categorized into four strata of different malaria transmission intensities based on the prevalence of malaria in school children aged 5–16 years: very low (<1%), low (1 - <5%), moderate (5 - <30%), and high (≥30%) (Thawer et al., 2020; Runge et al., 2022). Based on the WHO’s high-burden, high-impact initiative, the Tanzania National Malaria Control Programme (NMCP) targets these areas with tailored interventions to reduce the burden in high and moderate transmission areas and pursue elimination in regions with low and very low malaria transmission intensities (National malaria control program, 2020).

Until recently, most malaria genomic studies in Mainland Tanzania have generally analyzed small datasets and focused primarily on malaria high-transmission regions (Moser et al., 2020). This limited the geographic scope and could not provide a full exploration of the parasite genomic variations in many parts of Mainland Tanzania with varying transmission intensities. Currently studies in Tanzania have started to focus on generating information about the patterns of transmission and disease burden to support the ongoing initiatives to appropriately modify intervention strategies, inform local control programmes, and apply targeted interventions (Morgan et al., 2020; Moser et al., 2020). Leveraging both high-throughput genomic and epidemiological data, previous studies have generated baseline information on the patterns of parasite diversity, population structure, and drug resistance in high transmission regions on the Mainland (Morgan et al., 2020; Moser et al., 2020). Additional studies in Zanzibar, which has low transmission intensities and is currently implementing interventions for malaria elimination, have similarly documented patterns of parasite genetic diversity (Morgan et al., 2020; Holzschuh et al., 2023; Connelly et al., 2023). Most of these studies in both Mainland Tanzania and Zanzibar have reported varying patterns of parasite diversity (Morgan et al., 2020; Moser et al., 2020; Kulkarni et al., 2006) and signatures of selection (Morgan et al., 2020; Lyimo et al., 2024), and have provided evidence of importation of malaria to Zanzibar from Mainland Tanzania (Holzschuh et al., 2023; Connelly et al., 2023). While these studies have substantially advanced our understanding of malaria parasite populations in Tanzania and Zanzibar, they have not evaluated how different genetic metrics can be leveraged to infer local malaria transmission intensity or inform routine surveillance systems. Addressing this gap is essential to understand local malaria transmission intensity and to identify the utility of metrics in routine surveillance systems for informed local malaria control. Our study therefore aims to extend existing evidence by systematically assessing the utility of multiple genetic metrics for operational malaria surveillance across diverse transmission settings.

Recent studies undertaken in Tanzania and other endemic countries have leveraged Molecular Inversion Probes (MIPs), a high-throughput technique used to capture, amplify, and sequence short-targeted regions of the *P. falciparum* genome. MIPs data can be used to resolve different biological questions, such as the parasite’s genetic diversity and transmission dynamics (Moser et al., 2020; Aydemir et al., 2018; Verity et al., 2020). Previous work has shown that MIPs provide a relatively lower-cost and highly specific targeted sequencing approach due to their probe-based design (Moser et al., 2020; Aydemir et al., 2018; Verity et al., 2020). Importantly, beyond cost considerations, MIPs are also feasible for implementation in routine surveillance because they can be performed directly on commonly collected sample types such as dried blood spots (DBS) and require less complex laboratory infrastructure compared to whole-genome sequencing, workflow simplicity and short turnaround time, which all influence the suitability of MIPs for genomic surveillance programs in low-resource settings (Moser et al., 2020; Aydemir et al., 2018; Verity et al., 2020). MIPs have been successfully employed to study the spread of antimalarial drug resistance strains, parasite diversity and population structure in the Democratic Republic of Congo (Verity et al., 2020), Zambia (Fola et al., 2023a), Ethiopia (Fola et al., 2023b), Mainland Tanzania (Moser et al., 2020; Juliano et al., 2024; Ishengoma et al., 2024a) and Zanzibar (Morgan et al., 2020; Holzschuh et al., 2023; Connelly et al., 2023). Thus, their application in large-scale country-wide surveys, such as those undertaken by the project on molecular surveillance of malaria in Tanzania (MSMT) (Juliano et al., 2024), offers a practical approach to resolve genomic variations of the *P. falciparum* populations circulating in different transmission strata within Mainland Tanzania.

This study aimed to evaluate and identify the most informative genetic metrics for monitoring the genetic diversity of *P. falciparum* and to assess their spatial correlation with malaria transmission intensity across Mainland Tanzania. This was a cross-sectional study that collected dried blood spots (DBS) samples from symptomatic individuals at multiple health facilities in different regions of Mainland Tanzania. These sample types were chosen because symptomatic cases represent the majority of infections detected through routine health systems and therefore provide a practical and programmatically relevant source for molecular surveillance (Thawer et al., 2020). Using clinical parasite samples also enhances feasibility for large-scale implementation within endemic countries, as DBS are routinely collected, easy to store, and compatible with high-throughput genomic methods such as MIPs. This approach allows for consistent sampling across diverse transmission settings and supports the integration of genomic surveillance into ongoing national malaria control and elimination efforts. The correlations examined in this analysis reflect spatial differences across predefined transmission strata, rather than longitudinal changes. This study provides a broader picture of the parasite genetic diversity, which is critical for a better understanding of the contemporary parasite populations and transmission intensity across Mainland Tanzania. It supports the NMCP policy and decision-making as Tanzania progresses towards malaria elimination by 2030.

## Materials and methods

### Study sites and study design

**FIGURE 1:**
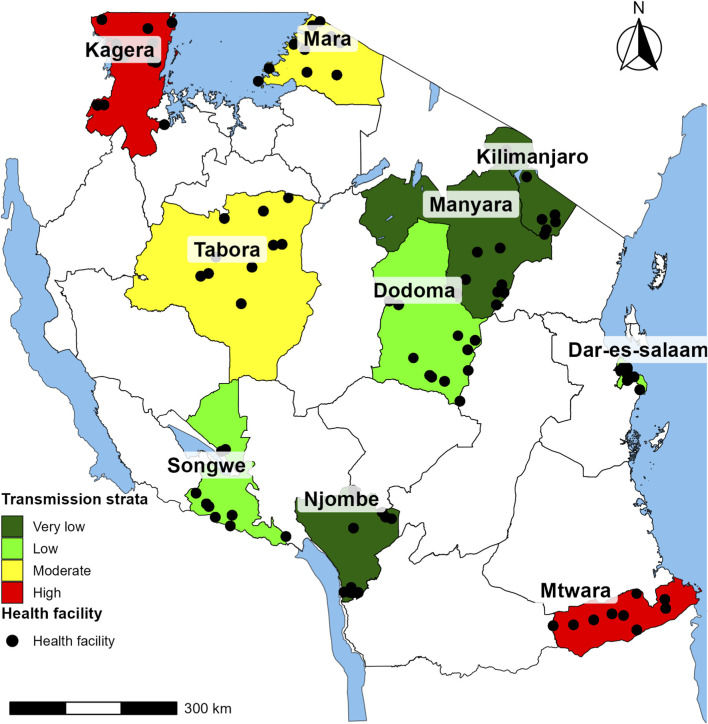
Map of Tanzania showing the 10 regions and malaria transmission strata color-coded as very low (dark green), low (light green), moderate (yellow), and high (red) (Thawer et al., 2020). Black dots represent the locations of health facilities which were covered by the study across the 10 regions (Juliano et al., 2024; Rogier et al., 2024; Popkin-Hall et al., 2024b).

Individual parasite samples and associated metadata for this study were obtained from a nationwide cross-sectional survey conducted by the MSMT project between February and July 2021. Parasite samples is defined as DBS collected from individuals who tested positive for *P. falciparum* by rapid diagnostic tests (RDTs). The methodology for selection of the study sites and parasite sample, as well as data collection procedures, have been described elsewhere (Rogier et al., 2023; Popkin-Hall et al., 2024a; Juliano et al., 2023). Briefly, a cross-sectional survey of symptomatic malaria patients was conducted in 100 health facilities (HFs) in 10 regions of Mainland Tanzania (Dar es Salaam, Dodoma, Kagera, Kilimanjaro, Manyara, Mara, Mtwara, Njombe, Songwe and Tabora) (Rogier et al., 2023; Popkin-Hall et al., 2024b). The HFs that participated in this study were conveniently selected from the NMCP sentinel sites and those that are hotspots of malaria. According to the 2020 malaria stratification by the NMCP, all 26 regions in Mainland Tanzania were grouped into four malaria transmission strata based on malaria prevalence in school children (aged 5–16 years), and the 10 regions sampled in this study were drawn from all four strata (Thawer et al., 2020) (Figure 1). Three regions were located in the very low malaria transmission stratum (Kilimanjaro, Manyara and Njombe), three in low transmission (Dar es Salaam, Dodoma and Songwe), two in moderate transmission (Mara and Tabora) and two regions in high transmission (Kagera and Mtwara).

### Sample collection, DNA isolation and MIP sequencing

Procedures for sample collection at the HFs were described in previous MSMT publications (Rogier et al., 2023; Popkin-Hall et al., 2024a; Popkin-Hall et al., 2024b). Briefly, febrile patients aged ≥6 months with symptoms of malaria such as fever or a history of fever were tested for malaria using RDTs. A total of 12,875 patients were screened for malaria using RDTs (either Abbott Bioline Malaria Ag Pf/Pan (Abbott Diagnostics Korea Inc., Korea) or Smart Malaria Pf/Pan Ag Rapid Test (Zhejiang Orient Gene Biotech Co. Ltd., China)) and 7,199 patients were confirmed to be RDT positive for *P. falciparum*. All DBS samples that were positive for *P. falciparum* by RDT were processed by taking 3 punches of 6 mm and placing them into 96 deep well extraction plates. Genomic DNA was extracted using the Chelex 100 + Tween20 protocol (Juliano et al., 2023; Walsh et al., 1991; Simon et al., 2021). Briefly, the punched DBS were incubated in 0.5% Tween 20 overnight at room temperature in the extraction plates to release parasites DNA from red blood cells followed by adding 1 mL of PBS and incubating at 4 °C for 15–30 min followed by a brief centrifugation to precipitate the DNA from other impurities. Then, a 1:2 solution of Chelex to sterile water (1.75 mL 20% Chelex + 3.5 mL PCR water) was added and incubated at 95 °C on a heat block for 10 min, followed by centrifugation for 5 min at high speed to pellet the Chelex and any degraded papers. The resulting supernatant was then transferred to new PCR plates followed by spinning for 10 min at 14,000 rpm ready for downstream assays. All *P. falciparum* RDT-positive samples were sequenced using MIPs, and the data generated were further analyzed with MIPtools (Supplementary Figure S1). MIP capture, sequencing, and PCR amplification were performed as described earlier (Moser et al., 2020; Aydemir et al., 2018; Verity et al., 2020; Fola et al., 2023a; Juliano et al., 2023) and targeted 1,832 single-nucleotide polymorphisms (SNPs) in the 14 chromosomes of the *P. falciparum* genome using IBC panel that is geographically informative to resolve the parasite genetic diversity. Briefly, all MIPs were pooled and undergone a 5′ phosphorylation using T4 polynucleotide Kinase (T4 PNK) before the capture reaction. 5 μL from each probe was pooled, followed by the addition of 91.6 μL of T4 PNK, 1145 μL of 10X T4 DNA ligase buffer, and 1053 μL of nuclease-free water. The mixture was well mixed and aliquoted into a 0.2 mL PCR plate, and incubated in a thermocycler at 37 °C for 45 min and 65 °C for 20 min. Capture reactions were carried out in 96-well plates, and each reaction contained 0.96 µL of 10X Ampligase buffer, 0.2 µL dNTPs, 0.4 µL MIP pool, 0.4 µL of Ampligase, 0.2 µL of Q5 polymerase and 5 µL of template DNA. The reactions were run in a thermal cycler at 95 °C for 10 min and 60 °C for 1 h and with a final hold at 4 °C. This was followed by the Exonucleases’ reaction which contained 0.8 µL of nuclease free water, 0.2 µL 10X Ampligase, 0.5 µL exonuclease I and 0.5 µL of exonuclease II and run in a thermal cycler at 37 °C for 1 h, 95 °C for 2 min and a final hold at 4 °C. Immediately after exonuclease treatment, barcoding PCR reaction mixtures were set up, and contained 5 μL of 5X Phusion reaction buffer, 5 μL of 5X MMC, 0.5 μL of 10 mM dNTPs, 0.25 μL of Phusion DNA polymerase, 1.25 μL of 10 μm forward primer, 1.25 μL of 10 μm reverse primers. The PCR conditions were 98 °C for 30 s, 98 °C for 10 s, 63 °C for 30 s, 68 °C for 30 s, 68 °C for 2 min for 38 cycles and a final hold at 4 °C. Then, all captured sequences were cleaned to remove unwanted adapters or primer dimmers using 1.2 x Ampure XP magnetic beads. Agarose gel electrophoresis was used to confirm capture success, and DNA libraries were pooled and purified using Ampure XP magnetic beads and then subjected to gel extraction and purification procedures using the New England Biolabs (NEB) Gel extraction kit (NEB Inc., Ipswich, MA, United States). Quality control was performed using a Qubit™ Fluorometer and a fragment analyzer. Libraries were denatured, diluted, and spiked with PhiX control before sequencing on the Illumina NextSeq 550 150 bp paired-end sequencing platform (Illumina, San Diego, CA inc., United States). Laboratory data generation and analysis were done at Brown University, United States (https://baileylab.org/) and the National Institute for Medical Research (NIMR) Genomics laboratory in Tanzania.

### Data management and bioinformatics analysis

**FIGURE 2:**
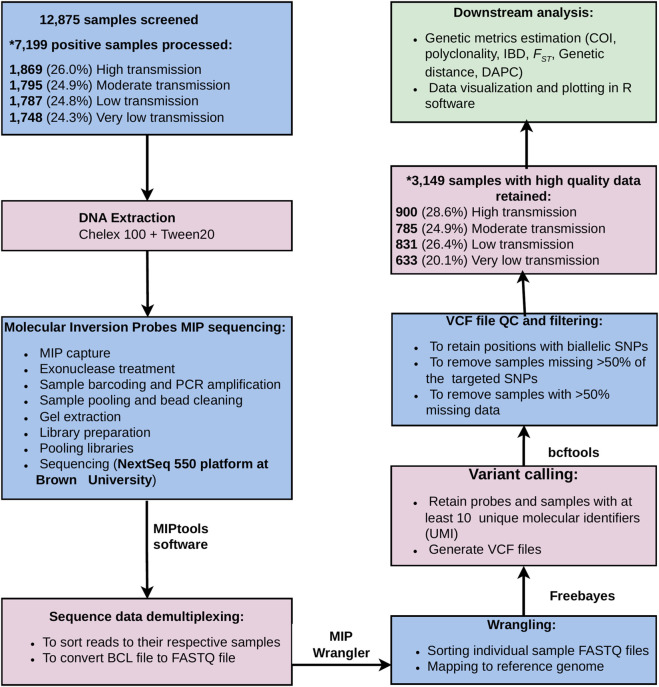
Flowchart illustrating a genomics workflow for sample processing, MIP sequencing, and bioinformatics and epidemiological data analysis. PCR = polymerase chain reaction, VCF = variant calling format, COI = complexity of infection, IBD = identity by descent, FST = fixation index, DAPC = discriminant analysis of principal component). *The proportions of parasite samples from regions located in the different strata of malaria transmission, which were sequenced (p = 0.07) and those which passed the filtering criteria and were used in the analysis (p = 0.14), were not significantly different.

Epidemiological and demographic data from study participants were collected at the sampled HFs using pen and paper questionnaires. After the survey, the data were double entered into Microsoft Access 2019 database (Version 2,302). The dataset was cleaned by removing duplicates, correcting mismatched information, eliminating parasite samples with missing variables, and creating new variables using the provided dictionaries, as outlined previously (Juliano et al., 2024; Gwarinda et al., 2021). The cleaned data were then merged with sequence data using the “dplyr” package (https://github.com/tidyverse/dplyr) in R version 4.5.1 for subsequent analysis. Data analysis included computation of descriptive statistics and estimation of genetic metrics within regions and across transmission strata. A one-way ANOVA was used to assess whether the mean number of successfully genotyped parasite samples differed significantly among the transmission strata in R version 4.5.1 software. The raw data generated using MIPs were demultiplexed using MIPtools software (https://github.com/bailey-lab/MIPTools), which are computationally suitable tools for MIP data processing and analysis of genomic data of *P. falciparum* (Moser et al., 2020). The data were further processed using MIP Wrangler software (https://github.com/bailey-lab/MIPWrangler), in which sequence reads sharing the same Unique Molecular Identifiers (UMIs) were collapsed to generate a single consensus. Each dataset was analyzed by mapping the sequence reads to the *P. falciparum* 3D7 reference genome using Burrows-Wheeler Aligner (BWA) (Chang et al., 2017; Early et al., 2019). Variant calling was done using the Freebayes (https://github.com/freebayes/freebayes) by retaining sequences with a minimum of 10 UMI per probe and sample. To retain good guality sequence data, quality control was performed by filtering only targeted SNPs using “bcftools” software version 1.13 (https://samtools.github.io/bcftools/bcftools.html). We filtered out parasite samples with >50% missing genotypes across all targeted SNPs and excluded SNPs with >50% missingness across all parasite samples, attributable to sequencing errors, low read depth, poor quality scores, or SNP genotyping failure. Multiallelic sites were removed, and only high-quality biallelic SNPs were retained for downstream analyses (Figure 2).

### Estimation of within-host genetic diversity

Within-host parasite genetic diversity was measured by calculating the complexity of infection (COI) and the proportion of polyclonal infections. The COI is defined as the number of genetically distinct parasite strains in a single infection (Gwarinda et al., 2021; Bei et al., 2018). The COI for each sample was calculated using a Markov Chain Monte Carlo categorical method for estimating COI likelihood (*THE REALMcCOIL*) version 1.3.1 as previous described (Chang et al., 2017). Briefly, the model was configured to consider a maximum of 25 clones per infection, the maximum permissible missing data for individual samples and SNP sites was established at 20%, and the analysis utilized 1,000 MCMC iterations with a burn-in period of 100 iterations. The proportion of polyclonal infections was defined as the number of parasite samples with COI >1 over the total number of parasite samples with successful genotypes, which was assessed in each region and transmission stratum. Polyclonality was also assessed by age, categorized into three groups: under-fives (aged <5 years), school children (5-<15 years) and adults (≥15 years).

The proportions of parasite samples with polyclonal infections in the regions sampled were compared to the mean annual test positivity rates of each region using the same year’s data, which was obtained from the District Health Information System 2 (DHIS2) of the Ministry of Health. The “ggplot2” package (https://github.com/tidyverse/ggplot2) was used to plot and visualize the distribution of COI per region, transmission strata and age group in R version 4.4.0.

### Analysis of parasite genetic relatedness

We used identity by descent (IBD) to detect fine-scale genetic relatedness among parasite populations. This is due to the fact that IBD measures the pairs of the genome shared between two parasite samples due to recent common ancestry, reflecting genetic relatedness between individual clones (Morgan et al., 2020). While shared ancestry may suggest recent connectivity between populations, IBD does not directly identify transmission events. This approach is particularly informative in high-transmission and heterogeneous settings where infections may arise from closely related parasite lineages. The pairwise IBD sharing among parasites was calculated for all parasite samples with monoclonal infection with COI = 1, (n = 1.878) in each region per transmission strata. We restricted the IBD analysis to parasite samples with COI = 1 (N = 1,878; 59.6% of samples) according to the MIPAnalyzer (https://github.com/mrc-ide/MIPanalyzer) methods that requires only monoclonal parasite samples to ensure accurate estimation of pairwise relatedness, as multi-clonal infections can inflate IBD values and complicate interpretation. We acknowledge that this restriction may bias the analysis toward lower complexity of infection; however, it provides a conservative and reliable measure of genetic relatedness among individual parasite clones. IBD was estimated using a maximum likelihood approach with the *inbreeding_mle* function from the *MIPanalyzer* package (https://github.com/mrc-ide/MIPanalyzer). In this study, IBD was calculated between all pairs of parasite samples within and among each region. IBD measures genetic relatedness between parasite pairs, ranging from 0 (unrelated) to 1 (clonal). In this study, we focused on only pairs with IBD ≥0.5 or ≥0.9 to aid interpretation. Pairs with IBD ≥0.5 were classified as highly related (sharing ≥50% of their genome), while pairs with IBD ≥0.9 were classified as near-clonal or clonal (sharing ≥90% of their genome), reflecting very recent common ancestry. These thresholds guided subsequent analyses of highly related parasites within and among regions. Pearson’s chi-square test was used to test for significant differences in IBD sharing among regions and transmission strata. To visualize parasite population cluster based on high IBD thresholds (IBD ≥0.5 and IBD ≥0.9), we applied t-distributed stochastic neighbor embedding (t-SNE) to a matrix of pairwise genetic distances derived from IBD estimates. Pairwise IBD values were converted to genetic distances as 1−IBD, and a symmetric distance matrix was constructed for all samples. t-SNE was performed using the R package Rtsne (version 0.17) with the distance matrix specified as input (is distance = TRUE) and a perplexity value of 30. The relationship between IBD and geographical distance was evaluated to investigate isolation-by-distance patterns. IBD networks were constructed at thresholds of ≥0.50 and ≥0.90 to identify highly related parasites and to assess patterns of inter-regional connectivity and local clustering.

### Parasite population structure and genetic differentiation

Parasite population structure and genetic differentiation analysis in this study utilized all parasite samples that were successfully sequenced (samples with good sequence data) (n = 3,149) independent of COI or IBD thresholds. For population genetic analyses, SNP genotypes were pseudo-haploidized by retaining a single allele per SNP per sample, ensuring comparability across monoclonal and polyclonal infections. Evidence of parasite genetic differentiation among the study regions was assessed by calculating the pairwise Wright’s fixation index (*F*_*ST*_) using the “hierfstat” version 0.5–11 package (https://github.com/jgx65/hierfstat) and genetic distance using the ‘adegenet’ (V.2.1.10) package (https://github.com/thibautjombart/adegenet) in R version 4.4.0. The *F*_*ST*_ reflects longer-term population divergence and provides a measure of broader scale structure (Gwarinda et al., 2023), complementing IBD’s focus on recent relatedness. However, the genetic distance matrix was used to summarize overall genomic similarity among parasite samples and regions. Genetic distances capture continuous gradients of relatedness and help visualize patterns that may not be apparent from discrete differentiation metrics like *F*_*ST.*_ To determine the population structure within and among *P. falciparum* populations in the regions, Discriminant Analysis of Principal Components (DAPC) (Gwarinda et al., 2023) was used as a clustering method to identify genetic groups. By maximizing between-group variation, DAPC allows detection of subtle structuring and evaluation of whether parasite clusters correspond to their geographical regions. This was performed using the “adegenet” (V.2.1.10) R package. Prior to analysis, all outlier values were removed to ensure that the results reflect true underlying genetic structure. Parasite samples were grouped by region, which served as the prior grouping factor. We retained the first 50 principal components (PCs) and 10 discriminant functions (DFs) for the analysis. The resulting DAPC plot visualizes the distribution of parasite samples across regions and highlights subtle population structure. The ggplot2 R package (v.3.4.0) was used to visualize the resulting DAPC plots.

### Association of the different genetic metrics with malaria transmission intensities

This analysis investigated the relationship between COI, polyclonality, IBD sharing and malaria transmission in R version 4.5.1 software. We conducted a series of statistical analyses to evaluate the relationships between key parasite genetic metrics and malaria transmission intensity, as well as demographic variables. To assess whether COI varies across transmission strata and with age, Kruskal–Wallis test using the R base was used to compare COI across the four transmission strata. Linear regression was applied to examine the association between COI and transmission strata as well as age by using the “lm” function in stats R package (version 4.5.1). A Spearman's rank correlation test was performed in R using the “cor.test” function from the stats package (version 4.5.1) to assess the relationship between mean COI and malaria test positivity rates (TPR) from the DHIS2 data. Significant associations were defined at p < 0.05. Logistic regression using generalized linear models with a binomial family “glm” function in stats package (version 4.5.1) was used to determine the association between polyclonal infections and transmission intensities and age groups and the results were presented as crude odds ratio (ORs) with 95% confidence intervals (CI), and statistic significance was determined at *p* < 0.05. Pearson’s correlation test was used to assess the association between the polyclonal infections and the malaria TPRs using a “chisq.test” function implemented in R (stats package, version 4.5.1). To assess whether pairwise IBD sharing differs across transmission strata, Pearson’s chi-squared test (*χ* ^2^) was used to test for differences in the distribution of IBD sharing across strata. The results were considered significant when p < 0.05.

### Sensitivity analysis using monoclonal infections (COI = 1)

Following the observation of low population differentiation and overlapping clusters in the *F*_*ST*_, genetic distance, and discriminant analysis of principal components (DAPC) analyses conducted on the full dataset, we performed a sensitivity analysis restricted to monoclonal infections (COI = 1). This was undertaken to evaluate whether the observed low genetic differentiation and overlapping population structure were influenced by the inclusion of polyclonal infections (COI >1).

We therefore repeated *F*_*ST*_ estimation, pairwise genetic distance calculations, and DAPC analyses using only monoclonal samples. This approach allowed direct comparison between results derived from the complete dataset (with samples that had COI >1 and C=1) and those restricted to samples with monoclonal infections (COI = 1), enabling assessment of the robustness of observed clustering patterns and differentiation metrics due to the inclusion of polyclonal infections.

## Results

### Dataset and site information

We screened a total of 12,875 patients from 100 HFs in 10 regions in Mainland Tanzania, and 7,199 (55.9%) patients were confirmed to be RDT positive for *P. falciparum*. All DBS samples that were positive for *P. falciparum* by RDT (n = 7,199) were sequenced using MIP, and after performing quality checks and filtering, 43.7% (n = 3,149/7,199) of the parasite samples had good quality data (Supplementary Table S1; Supplementary Figure S1), and 55.2% (n = 1,011/1,832) of all the targeted SNPs had good quality. The retained SNPs were distributed across the 14 chromosome of *P. falciparum* genome (Supplementary Figure S2). These parasite samples were considered to have been successfully sequenced and were retained for downstream analysis. The proportions of parasite samples with genotyping success (n = 3,149) from the four malaria transmission strata were not significantly different (p = 0.14), with 20.1% (n = 633/3,149) from regions located in the stratum with very low, 26.4% (n = 831/3,149) from low, 24.9% (n = 785/3,149) from moderate, and 28.6% (n = 900/3,149) from regions located in the high transmission stratum. The majority of the parasite samples with high quality data were from the priority regions of Kagera (18.3%, n = 576/3,149), Tabora (13.0%, n = 410/3,149) and Mara (10.6%, n = 334/3,149) while the Kilimanjaro region had fewer parasite samples (3.6%, n = 114/3,149). The number of parasite samples that passed quality filters and were successfully sequenced was not uniform across regions. However, the number of parasite samples that were successfully genotyped did not differ significantly among the four transmission strata (P = 0.12). Differences in malaria burden, sample availability, and sequencing success resulted in an uneven distribution of high-quality parasite samples across the study regions and transmission strata (Supplementary Figure S1; Supplementary Table S1).

### Variations in the complexity of infection by transmission intensity and other variables

**FIGURE 3:**
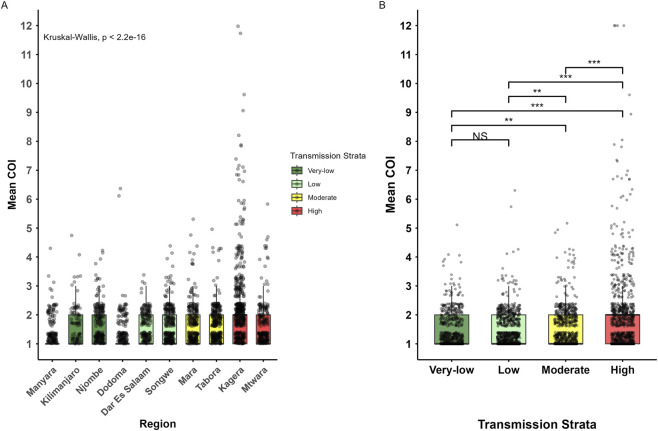
Distribution of mean complexity of infection (COI). Boxplots show the distribution of mean COI for parasite samples collected in each region **(A)** and in the different transmission strata **(B)** with points representing individual infection. Boxplots depict the median (center line), interquartile range (IQR, upper and lower quartiles), whiskers, and outliers (points). Colors correspond to transmission strata (very low = dark green, low = light green, moderate = yellow, high = red). Statistical significance was assessed using a Kruskal–Wallis test. Higher COI suggests greater within-host parasite diversity and may indicate higher local transmission. Significance levels: p < 0.001, **p < 0.01, *p < 0.05, NS = Not significant.

Within-host genetic diversity based on COI showed that over half of the parasite samples (59.6%, n = 1,878/3,149) carried monoclonal infections (COI = 1), and 40.4% (n = 1,271/3,149) had polyclonal infections (COI >1). The country-wide mean COI was 1.5, with the highest mean COI in Kagera (COI = 2.1, range = 1–22) and the lowest mean COI in Manyara (COI = 1.3, range = 1–4) (Figure 3A; Supplementary Table S2). Among the parasite samples with polyclonal infections, 80.3% (n = 1,020/1,271) had COI = 2, 11.2% (n = 142/1,271) had COI = 3, and 8.3% (n = 105/1,271) had COI ≥4. Only a small fraction of parasite samples (0.3%; n = 4/1,271) had COI ≥10.

The mean COI was significantly higher in high (COI = 1.8, range = 1–22) and moderate (COI = 1.5, range = 1–6) transmission strata compared to low and very low transmission (p < 0.001), but it was similar in the regions within low (COI = 1.4, range 1–5) and very low transmission strata (COI = 1.4, range = 1–5) (p = 0.92) (Figure 3B). The mean COI was statistically different among regions and transmission strata (p < 0.001). Using the high transmission stratum as a reference group, the mean COI significantly decreased in other transmission strata, with the coefficient = −0.51 (95% CI: 0.61 - -0.41, p < 0.001) in very low statum, coefficient = −0.52 (95% CI: 0.61 - -0.42, p < 0.001) in low and the coefficient = −0.43 (95% CI: 0.53 - -0.33, p < 0.001) in moderate transmission strata. A Spearman rank correlation test was performed to assess the relationship between mean COI and malaria TPRs from the DHIS2 data. Although a moderate positive correlation was observed (rho = 0.375), this association was not statistically significant (p = 0.286). The differences in mean COI among patients of different age groups were statistically significant (p < 0.001), with higher mean COI (1.6) among school children, followed by under-fives (COI = 1.5), and the lowest (COI = 1.4) was in adult patients. The difference in mean COI was statistically significant between under-fives and adult patients (p = 0.003) and between school children and adult patients (p = 0.03). However, there was no significant difference in mean COI between under-fives and school children (p = 0.30) (Supplementary Figure S3). The mean COI was similar among female (COI = 1.6) and male (COI = 1.5) participants (p = 0.40) (Supplementary Figure S4).

### Spatial heterogeneity of polyclonal infections and transmission intensity

**FIGURE 4:**
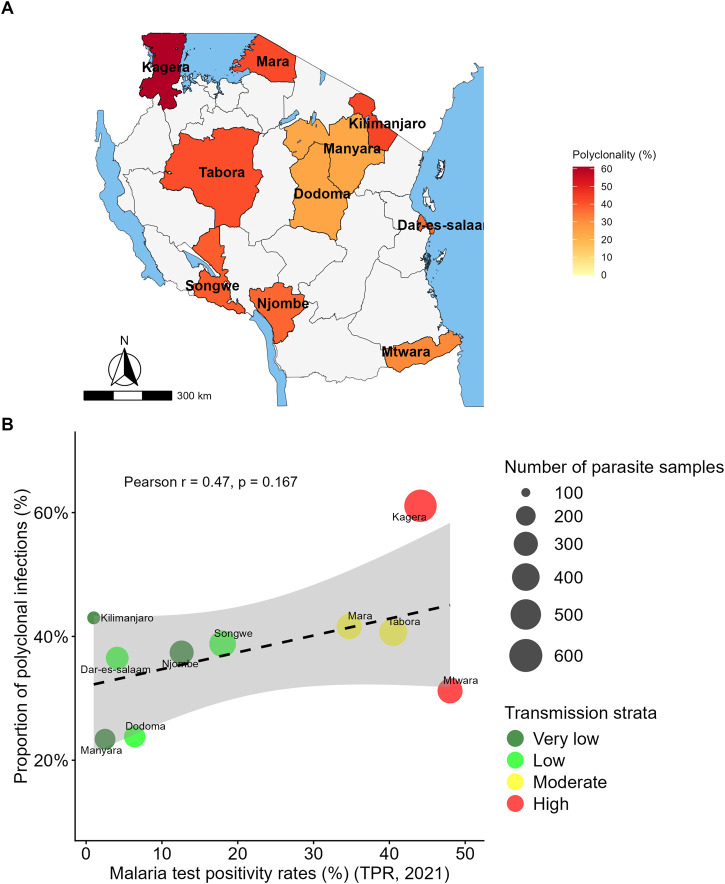
**(A)** A map of Tanzania showing the spatial heterogeneity in the proportion of polyclonal *P. falciparum* infections across regions with color intensity increasing as the proportion of polyclonal infections increase. **(B)** A scatter plot depicting the relationship between regional proportions of polyclonal infections and malaria TPRs. Each point represents a region, with size proportional to the number of parasite samples analyzed. Pearson’s correlation coefficient (r) and the associated p-values are shown, illustrating the association between the proportion of polyclonal infections and malaria TPRs. Circles are colored based on regional malaria transmission intensity (red = High, yellow = Moderate, light green = Low, dark green = Very low transmission intensity).” TPR = test positivity rate by rapid diagnostic tests - RDTs; Data used was from DHIS2 in 2021.

**FIGURE 5:**
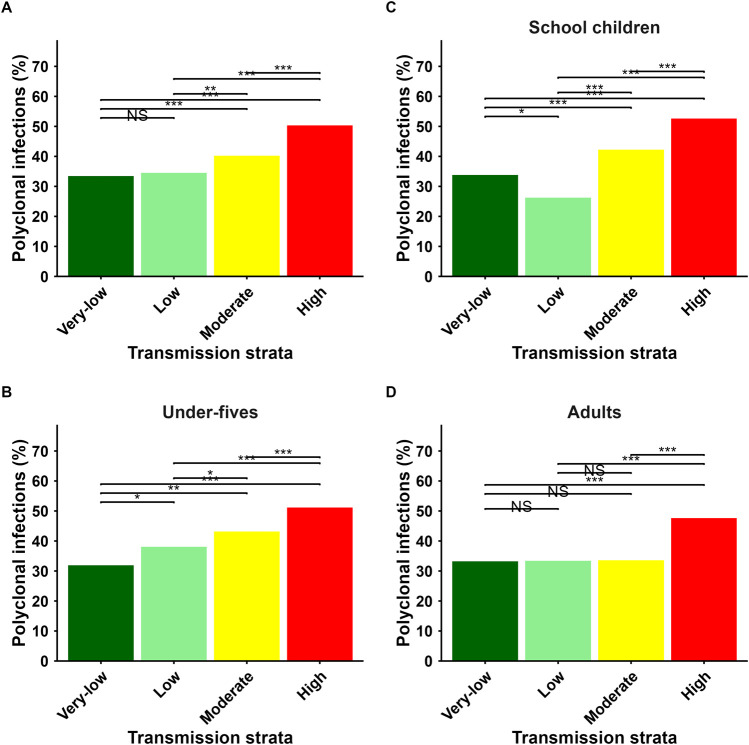
Bar charts showing the overall proportion of polyclonal infections across different transmission strata **(A)**. Using high transmission strata as a reference category, the proportion of polyclonal infection was statistically different among all transmission strata. The proportion of polyclonal infections across transmission strata differed by age groups (**(B)** = under-fives, **(C)** = School children and **(D)** = Adults). The proportion of polyclonal infections was high in high transmission stratum across all age groups. Transmission strata on the x-axis are colored consistently with the NMCP malaria transmission intensity. ***p < 0.001, **p,0.01, *p < 0.05, NS = Not significant.

The countrywide proportion of polyclonality (COI >1) was 40.4% (n = 1,271/3,149). Among all regions, the highest proportion of parasite samples with polyclonal infections was in Kagera (61.3%, n = 353/576), and the lowest was in Manyara (23.4%, n = 52/222) as presented in Figure 4A and in Supplementary Table S2. The proportion of parasite samples with polyclonal infections in each region was relatively similar to the regional malaria TPRs based on Tanzania DHIS2 2021 data, except for Kilimanjaro, which had fewer sequenced parasite samples. Regional proportion of polyclonal infections was positively correlated with malaria TPRs (Pearson r = 0.47, 95% CI = −0.30 to 0.82, p = 0.17) although this was not statistically significant (Figure 4B). The proportion of parasite samples with polyclonal infections was significantly higher in the high (50.4%; n = 454/900) and moderate transmission strata (40.4%; n = 317/785), compared to low (34.7%; n = 288/831) and very low strata (33.5%; n = 212/633, p < 0.001) (Figure 5A). Using the high transmission as a reference, the odds of polyclonal infections were significantly lower in moderate (aOR = 0.67; 95%CI: 0.55–0.81; p < 0.001), low (aOR = 0.52; 95%CI: 0.43–0.63; p < 0.001) and in very low transmission strata (aOR = 0.49; 95%CI: 0.40–0.61; p < 0.001). The overall proportion of polyclonal infections was 43.9% (n = 500/1,138) in under-fives 41.0% (n = 327/798) in school children and 36.6% (n = 444/1,213) among adult patients. The odds of polyclonal infections were lower in adult patients compared to school children (aOR = 1.20; 95% CI: 1.00–1.45; p = 0.048) and under-fives (aOR = 1.36; 95% CI: 1.15–1.60; p < 0.001). The proportion of polyclonal infections was statistically significantly different among under-fives and school children across all transmission strata (Figures 5B,C). Among adults, the proportion of polyclonal infections was similar in very low, low and moderate strata but statistically different in high stratum compared to others (Figure 5D). Across all age groups, the proportion of polyclonal infections was higher in high transmission stratum compared to other strata (Figures 5B–D). The odds of polyclonal infections were not significantly different among males and females (aOR = 0.89; 95% CI: 0.77–1.03; p = 0.11) (Supplementary Figure S5).

### Parasite relatedness and transmission intensity

Overall, the majority of pairwise IBD values across parasite samples were low (IBD <0.1), indicating weak genetic relatedness among most parasite pairs (Supplementary Figure S6, S7). Most pairwise comparisons fell within the lowest IBD range (0–0.1), accounting for approximately 98.2% of all comparisons, while only a small fraction exceeded the threshold of IBD ≥0.5. Highly related parasite sample pairs (IBD ≥0.5) ranged from 0.01% in Tabora to 0.91% in Dar es Salaam, while near-clonal pairs (IBD ≥0.9) ranged from 0.00% in Tabora to 0.61% in Dodoma. Overall, only a small subset of parasite pairs exhibited high or near-clonal relatedness, appearing as rare outliers rather than the norm across all regions.

Among parasite sample pairs meeting the predefined relatedness thresholds, the average countrywide IBD was 0.89 for pairs sharing ≥50% of their genome (half-siblings/highly related) and 0.98 for pairs sharing ≥90% (near-clonal or clonal). Highly related and near-clonal pairs were rare across all sites. Low-transmission regions (Dar es Salaam, Dodoma, Songwe) exhibited slightly higher proportions of highly related pairs (IBD ≥0.5: 0.1–0.91; and IBD ≥0.9: 0.07–0.61) compared to high-transmission regions (Kagera, Mtwara: IBD ≥0.5: 0.1%–0.44%; IBD ≥0.9: 0.08%–0.34%). Although highly related and near-clonal parasite pairs were uncommon overall, they occurred more frequently in low-transmission settings.

**FIGURE 6:**
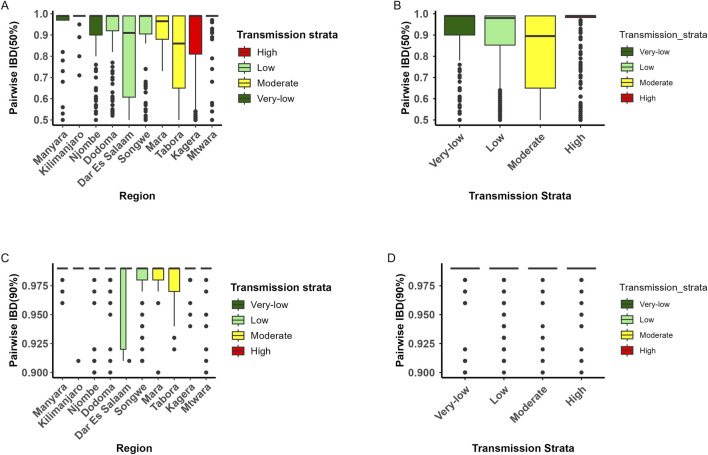
The distribution of pairwise identity-by-descent (IBD) for parasite pairs grouped by transmission strata, ordered from very low to high. The boxplots depict the median (center line), interquartile range (IQR, upper and lower quartiles), whiskers, and outliers (points). Colors represent malaria transmission strata (very low = dark green, low = light green, moderate = yellow, high = red). Panel shows: **(A)** pairwise IBD for parasites sharing ≥50% of their genome segments (half-siblings) per regions, **(B)** pairwise IBD per transmission strata, **(C)** pairwise IBD for parasites sharing ≥90% of their genome segments (full-siblings) per region and **(D)** shows pairwise IBD for parasites sharing ≥90% of their genome segments (full-siblings) per transmission strata. The decline in IBD values from parasite pairs sharing ≥50% to those sharing ≥90% of their genome reflects decreasing genetic similarity, consistent with higher rates of outcrossing among genetically distinct parasites.

At the ≥50% IBD threshold, genetic relatedness within regions (Figure 6A) and across transmission strata (Figure 6B) was relatively higher but not statistically significant, and decreased further at the ≥90% threshold within regions (Figure 6C) and across transmission strata (Figure 6D). These estimates summarize the degree of genetic similarity within the subset of highly related parasite pairs and do not represent the overall distribution of pairwise relatedness across all samples, which is shown in Supplementary Figure S6.

**FIGURE 7:**
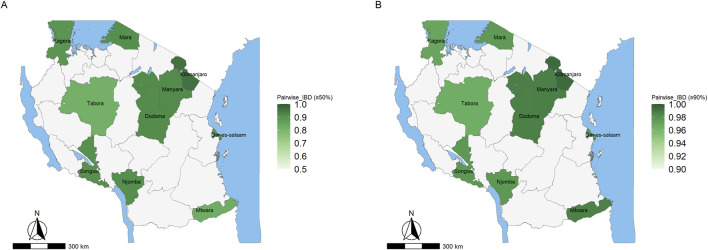
Map of Tanzania showing the average pairwise identity-by-descent (IBD) among parasite pairs sharing ≥50% of their genome segments (highly related) - Panel **(A)**, while Panel **(B)** displays the average IBD among parasite pairs sharing ≥90% of their genome segments (near-clonal or clonal). In both panels, regions are shaded in varying intensities of green, with darker green indicating higher average IBD and greater genetic relatedness.

The regional average IBD among highly related parasite pairs (IBD ≥0.5) ranged from 0.81 in Tabora to 0.96 in Kilimanjaro (Figure 7A), while near-clonal pairs (IBD ≥0.9) ranged from 0.96 in Tabora to 0.99 in Kilimanjaro (Figure 7B). However, no region showed exceptionally elevated overall IBD relative to others, indicating that while certain parasite pairs were highly related or near-clonal, the majority of pairs remained weakly related. The overall distribution of pairwise IBD across all parasite pairs is shown in Supplementary Figure S7, providing the context for these conditional averages. The t-SNE visualization of IBD-derived genetic distances did not reveal distinct clustering of parasite samples by transmission strata Supplementary Figure S8. Instead, samples from high, moderate, low, and very-low transmission regions largely overlapped in the two-dimensional embedding, indicating substantial genetic similarity across strata. Overlapping clusters across strata indicate limited genetic structuring and shared ancestry among highly related parasites across transmission settings. However, we analyzed the effect of geographical distance on genetic relatedness and found that IBD decays as the distance between samples increases, consistent with an isolation-by-distance pattern (Supplementary Figure S9). At a high relatedness threshold (IBD ≥0.50), some inter-regional connections were detectable, suggesting occurrence of parasite movement across geographic areas (Supplementary Figure S10A). Also, fragmentation was observed at IBD ≥0.90 threshold, possibly indicating that highly related parasites were largely confined within regions, with minimal evidence of extensive long-distance connectivity (Supplementary Figure S10B). The absence of a large, highly connected component argues against widespread clonal expansion or dominance of a single lineage.

### Parasite genetic differentiation and population structure

**FIGURE 8:**
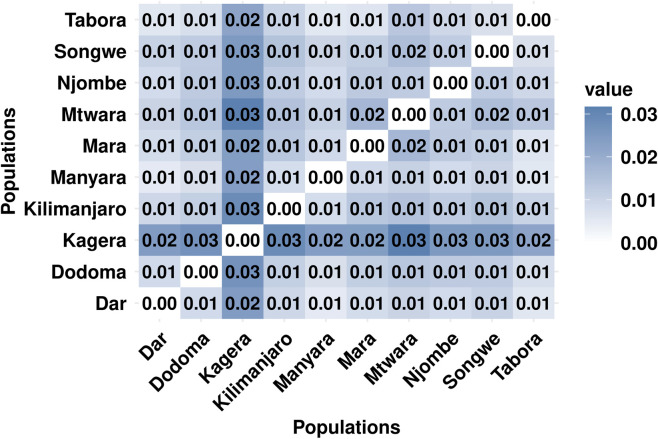
The heatmap showing pairwise genetic distances among parasite populations from 10 regions of Mainland Tanzania, based on the genome wide genetic distance matrix with darker shades indicating lower genetic similarity and lighter shades indicating high genetic similarity in parasite populations.

**FIGURE 9:**
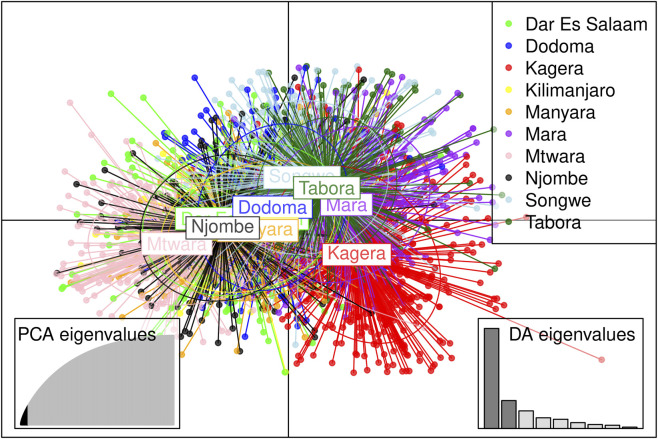
Discriminant Analysis of Principal Component (DAPC) plot showing the population structure of *P. falciparum* across Mainland Tanzania with different colours representing parasites for each region. The bar chart on the bottom left represents the eigenvalues, which show genetic variations explained by each principal component. The bar chart on the bottom right shows the contribution of the discriminant axes (DA) to the cluster separation, with the first two axes accounting for most of the explained variations.

Based on the pairwise *F*_*ST*_ *,* there was very little to no genetic differentiation among parasite populations in all regions, with *F*_*ST*_ values ranging from 0 to 0.006 among the regions (Supplementary Figure S11). The *F*_*ST*_ results showed that parasite populations from regions with a high geographical distance between them had higher *F*_*ST*_ values compared to closer regions, suggesting that there was some level of genetic differentiation, although the values did not reach a significant cut-off of *F*_*ST*_ ≥0.05. Genetic distance analysis also revealed very little to no genetic distance among parasites across all regions, with values ranging from 0 to 0.03 (Figure 8). The DAPC analysis revealed overlapping clusters (Figure 9) among parasite populations with no clear distinct parasite population structure formed. Parasite populations across regions clustered together, showing within-region parasite genetic similarity. Different parasite populations formed overlapping clusters, indicating limited genetic diversity among parasites circulating across transmission strata within Mainland Tanzania (Supplementary Figure S12).

### Sensitivity analysis using monoclonal infections (COI = 1)

A sensitivity analysis restricted to monoclonal infections (COI = 1) yielded results consistent with those obtained from the full dataset. The *F*_*ST*_ and genetic distance estimates remained low (Supplementary Figure S13), and DAPC analysis continued to show substantial regional overlapping clustering, with no evidence of increased population structure following exclusion of polyclonal infections (Supplementary Figure S14).

## Discussion

This was the largest *P. falciparum* genomic study of its kind in Mainland Tanzania, which evaluated various genetic metrics using the largest genomic dataset collected from 10 regions across four malaria transmission strata. It aimed to evaluate and identify the most informative genetic metrics for monitoring the genetic diversity of *P. falciparum* and its correlation with changes in malaria transmission intensities in Mainland Tanzania.

The findings offered insights into the usefulness of COI, proportion of polyclonality, and IBD in resolving local transmission intensity in regions with different endemicity levels, utilizing RDT-positive parasite samples processed rapidly with MIP sequencing. This study provides crucial evidence on *P. falciparum* genomic diversity and the informativeness of genetic metrics for malaria surveillance in Tanzania’s elimination efforts. Despite a large dataset analyzed (n = 7,199 *P. falciparum* positive by RDT), the success rate was lower, and only 43.7% of the parasite samples were retained for downstream analyses after filtering, which was not unexpected. This may be due to the fact that HRP2-detecting RDTs are known for high false positivity rates due to antigen persistence (Rogier et al., 2024), and some positive parasite samples had low parasitaemia that may be hindering sequencing success, particularly in low transmission settings (Early et al., 2019). In addition, MIP sequencing utilizes probes which are applied to raw parasite samples before amplification, making this approach have a low detection and success rate compared to the methods that work with amplified PCR products. The success rate is expected to be low in parasite samples with varying levels of parasitaemia, such as those that were analyzed in this study, since they were only *P. falciparum* RDT positive samples with unknown levels of parasitaemia. However, parasite samples retained after filtering were not uniform across different regions. Despite these limitations, MIP sequencing remains a fast and cost-effective method for large-scale analysis and rapid results.

We observed high within-host parasite diversity, as reflected by elevated COI and a high proportion of polyclonal infections. In high-transmission settings, frequent superinfection and co-transmission increase the probability that genetically distinct parasite clones are taken up by the same mosquito, thereby elevating the likelihood of outcrossing. This outcrossing occurs during the parasite’s obligatory sexual stage and generates novel genetic combinations through meiotic recombination, contributing to higher population-level genetic diversity relative to lower-transmission areas (Watson et al., 2021; Neafsey et al., 2021; Wong et al., 2018). In contrast, low and very low transmission areas had significantly lower COI and polyclonality, likely due to reduced immunity resulting in earlier treatment of symptomatic cases, further limiting the chance for multiple strain infections (Moser et al., 2020; Ishengoma et al., 2024b) in these areas. While the correlation between COI and polyclonal infections with malaria TPRs (from the DHIS2 data) suggested that regions with higher transmission tended to have a higher COI and proportion of polyclonal infections, the relationship was not strong enough to reach significance, likely reflecting uneven number of successfully sequenced parasite samples across regions and potential underlying epidemiological differences among sampled populations. These factors may influence COI and polyclonality independently of the regional malaria TPRs. Previous reports (Moser et al., 2020; Thawer et al., 2020; Runge et al., 2022) have demonstrated a strong association between mean COI and polyclonality with transmission intensity, highlighting the potential of COI and polyclonality as robust metrics for tracking malaria transmission dynamics and evaluating intervention impact in Mainland Tanzania and beyond. Kilimanjaro, despite being in a very low transmission stratum, exhibited a high mean COI (1.52) and a 42.9% rate of polyclonal infections. Although the number of parasite samples meeting filtering criteria from this region was limited, the data suggests substantial malaria parasite diversity, likely due to multiple strain infections. This contrasts with the typical scarcity of multiple parasite infections in such low transmission settings. The elevated diversity in Kilimanjaro could be attributed to the importation of genetically distinct parasite strains by travelers and migrants from moderate and high transmission areas. Parasites from Kilimanjaro (Figure 9) showed strong genetic links to parasites from other regions and clustered closely with them in the DAPC analysis. As a tourist hub with significantly high human movements, Kilimanjaro’s high genetic diversity while it is located in a low malaria setting is consistent with findings that importation of diverse parasite strains contributes to this phenomenon. This has been highlighted in different studies conducted in Zanzibar (Morgan et al., 2020; Holzschuh et al., 2023; Connelly et al., 2023), Zambia (Fola et al., 2023a) and the Kingdom of Eswatini (Roh et al., 2019).

The mean COI and the proportion of polyclonal infections were not associated with sex. However, there were significant differences in mean COI among age groups, with the highest mean COI among under-fives and school children compared to adults across all transmission strata. The high transmission stratum had higher proportion of polyclonal infections across all age groups (Figure 5). Under-fives and school children were the only age groups in which there was a consistent positive relationship between the proportion of polyclonal infections and transmission strata. The proportion of polyclonal infections among adults were similar in very low, low and moderate transmission strata and different in high transmission stratum as compared to other strata in the same age group. However, school children were highlighted to be the contributors of high COI in the studied areas. This is a semi-immune group that often do not become ill enough to seek treatment and thus have a higher rate of superinfections due to the stacking of long-lasting infections, which would otherwise be cleared by effective antimalarials. High mean COI and *P. falciparum* genetic diversity among under-fives and school children have been reported in most endemic countries, including Zambia (Fola et al., 2024), Kenya (Kimenyi et al., 2022), Tanzania (Kisambale et al., 2025) and Cameroon (Metoh et al., 2020). A high rate of polyclonal infections among younger children could be influenced by variability in their level of acquired immunity (Boyle et al., 2024). In malaria-endemic areas, immunity against malaria develops with age due to exposure to malaria infections that depend on the level of transmission intensity (Isiko et al., 2024). Underdeveloped immunity can allow different strains of parasites to be detected in an infection, while fully developed immunity can help to clear certain strains, leading to lower complexity of infection (Steinhardt et al., 2020). In addition, recent studies from country-wide surveys have shown that the prevalence of malaria infections in most parts of Tanzania is higher among school children and under-fives in some areas (Popkin-Hall et al., 2024a; Popkin-Hall et al., 2024b; Mandai et al., 2024; Challe et al., 2024; Chacha et al., 2025). This could explain the high complexity of infection observed in this study in these groups. Additionally, a country-wide analysis of subpatent infections has reported a higher prevalence of such infections in under-fives and school children, further supporting the observed high complexity of infection in these children (Seth et al., 2025). Thus, future studies will be needed to further explore the causes of the high complexity of infection in children and how this pattern responds to interventions currently directed to these groups.

Analysis of parasite relatedness revealed that, at IBD thresholds (≥0.50), some inter-regional connections are detectable, reflecting occasional parasite movement across geographic areas. However, at higher thresholds (IBD ≥0.90), the network is highly fragmented, indicating that near-clonal parasites are largely confined to small, localized clusters. Highly related parasites were predominantly clustered within regions, suggesting that transmission is largely localized, with limited evidence of widespread clonal expansion or long-distance dispersal. The absence of a large, highly connected component further argues against dominance by a single expanding lineage. Sensitivity analyses restricted to monoclonal infections confirmed that these patterns are robust and not driven by polyclonal averaging, indicating that the observed low differentiation reflects genuine gene flow and transmission connectivity rather than analytical artifacts.

At the population level, multivariate analyses reinforced these patterns. t-SNE of parasite pairs with IBD ≥0.5 showed extensive overlap of groupings across regions and transmission strata, with highly related parasites interspersed rather than forming distinct regional or transmission-specific clusters. These findings were mirrored by Discriminant Analysis of Principal Components (DAPC), which demonstrated substantial overlap among parasite populations across most regions, supporting limited population structure and widespread gene flow in Mainland Tanzania. Consistently, pairwise FST and genetic distance estimates were uniformly low and largely non-significant, reflecting long-term connectivity and shared genetic diversity rather than recent, localized transmission events.

Geographical distance further explained patterns of relatedness: IBD decayed as the distance between samples increased, consistent with an isolation-by-distance pattern, while remaining high within regions, indicating that near-identical parasites are largely confined to local areas. Across transmission strata and regions, highly related or near-clonal parasites (IBD ≥0.5 or ≥0.9) were generally rare, likely reflecting frequent outcrossing between genetically distinct clones during the mosquito stage and the resulting genetic reshuffling. Taken together, individual- and population-level analyses provide a coherent picture of malaria parasite population structure in the study regions: gene flow and connectivity occur broadly at the population level, while micro-epidemiological expansion remains highly localized, creating a landscape characterized by regional mixing interspersed with small, highly related clusters.

The consistently low *F*_*ST*_ and genetic distance estimates and extensive overlap observed in DAPC analysis indicate limited population differentiation across regions. Importantly, restricting the analyses to monoclonal infections (COI = 1) yielded comparable results, suggesting that the observed weak structure is not an artefact of polyclonal infections or allelic averaging. The persistence of overlapping clustering following exclusion of polyclonal samples supports the robustness of the inference that parasite populations across regions are highly connected. These findings are consistent with substantial gene flow and ongoing parasite movement between transmission settings, potentially facilitated by human mobility, vector movement, and shared transmission corridors. In such epidemiological contexts, high connectivity can homogenize allele frequencies across regions, thereby reducing measurable genetic differentiation despite geographic separation. The absence of distinct genetic clustering suggests that regional parasite populations do not represent isolated transmission foci but rather form part of a broadly interconnected population. Given the heterogeneity in transmission intensity across Mainland Tanzania, the lack of pronounced genetic structuring suggests that parasite movement may counterbalance local ecological differences, maintaining genetic homogeneity across epidemiological strata.

Overall, the combined evidence from IBD, t-SNE, DAPC, *F*_*ST*_, and genetic distance analyses indicates minimal genetic differentiation and extensive connectivity among *P. falciparum* populations across Tanzania, despite substantial heterogeneity in transmission intensity. The lack of clear clustering by transmission strata suggests extensive gene flow among parasite populations across regions or shared circulating lineages highlighting that transmission intensity alone does not fully explain patterns of genetic similarity at the national scale. Importantly, these metrics are not contradictory but reflect processes operating at different biological and temporal scales: IBD captures rare, recent transmission events between individual parasites, whereas *F*_*ST*_, genetic distance, and multivariate analyses reflect longer-term population connectivity and shared ancestry. Highly related parasite pairs were uncommon and did not form discrete regional clusters, supporting a model of widespread parasite mixing rather than sustained localized clonal expansion. These findings underscore the importance of considering national-scale parasite connectivity when designing malaria control and elimination strategies, particularly in low transmission settings where imported infections may play a disproportionate role in sustaining transmission.

This country-wide genomic analysis has revealed high genetic diversity, spatial patterns of parasite relatedness, and limited population structure among *P. falciparum* populations in Mainland Tanzania. The mean COI and the proportion of polyclonal infections revealed a high correlation with transmission intensities and can be potentially used to assess trends and patterns of malaria transmission and evaluate the impact of malaria interventions in Mainland Tanzania. However, further validation is needed to link these measures to specific interventions. They can be integrated into routine surveillance to resolve malaria transmission dynamics and assess the effectiveness of interventions. Also, this study proves the beneficial use of a molecular surveillance approach that covered all four transmission strata, providing a broader picture of the *P. falciparum* genetic diversity and current transmission intensity. As the country experiences high to very low transmission intensities, further research and analysis covering the entire country should be done to understand the genetic characteristics of circulating parasites and develop appropriate control interventions for each stratum across the country.

## Limitations of the study

This study used data from cross-sectional surveys to study different genetic metrics of parasites circulating in different areas of Mainland Tanzania during that sampling time. These results cannot reveal any temporal nature or patterns of parasite populations in the country. Thus, longitudinal surveys with data collection covering different seasons are recommended in future studies.

In addition, sampling techniques were not random because surveys were done in HFs that had sufficient numbers of cases and were capable of attaining the required sample size. However, *P. falciparum* analyzed were based on detection by RDTs only, which these tests have a major limitation of high false positivity rates due to the persistence of HRP2 antigens after treatment and recent past infections. Also, the sample size was not evenly distributed across the regions. Some regions contributed substantially more parasite samples than others due to differences in malaria burden, HF catchment sizes, and operational constraints during sample collection. These variations may influence regional comparisons of genetic metrics such as COI, IBD, and parasite diversity. Therefore, future studies with more balanced sampling would help strengthen these comparisons. However, we did not include drug resistance or other adaptation-related loci in the analyses. Consequently, we cannot assess whether shared selection pressures contributed to the observed limited population structure. Future studies incorporating genome-wide selection scans could help clarify the role of specific loci in shaping parasite connectivity.

## Data Availability

The genomic data generated in this study are publicly available through the European Nucleotide Archive (ENA) and the European Variation Archive (EVA). The study is registered under PRJEB109772 (ERP190552), titled MMS IN TANZANIA. Individual sample accessions range from ERS29452777 to ERS29455925. Variant call format (VCF) files generated from MIP genotyping are available under submission ERA35989591. Sample metadata and additional files to reproduce the analyses are available at our GitHub repository: https://github.com/dativapereus/MSMT-manuscripts.
