# Supplementary material for: Genetic metrics decode Plasmodium falciparum diversity: complexity of infection, parasite connectivity, and transmission intensity in Mainland Tanzania’s diverse regions
Source: Front Genet. 2026 Apr 29;17:1695073. doi: 10.3389/fgene.2026.1695073 (PMC13168215; doi:10.3389/fgene.2026.1695073)
Supplement: Supplementary file 1 [file Supplementaryfile1.pdf]

SUPPLEMENTARY FILES

Table S1. Summary of samples collected in each of the study regions stratified by transmission strata

Transmission strata	Region	Total Positivity Rates (TPR) - DHIS2 2021 (%)	Negative RDT (n)	Positive RDT (n)	RDT Malaria positivity rate (%)	Samples with good quality data	Samples with good quality that were used in the final analysis (%)
Very-low	Kilimanjaro	1.8	2654	615	615/3269 (18.8)	114	114/615 (18.5)
	Manyara	3.1	292	562	562/854 (65.8)	222	222/562 (39.5)
	Njombe	12.2	283	571	571/854 (66.9)	297	297/571 (52)
**Sub-total**			**3229**	**1,748**	**1,748/4,977 (35.1)**	**633**	**633/1,748 (36.2)**
Low	Dar-es-salaam	2.2	241	572	572/813 (70.4)	266	266/572 (46.5)
	Dodoma	4.6	417	594	594/1,011 (58.8)	235	235/594 (39.6)
	Songwe	7.6	269	621	621/890 (69.8)	371	371/621 (59.7)
**Sub-total**			**927**	**1,787**	**1,787/2,714 (65.8)**	**872**	**872/1,787 (48.8)**
Moderate	Tabora	39.9	233	868	868/1,101 (78.8)	410	410/868 (47.2)
	Mara	34.5	437	927	927/1,364 (68.0)	334	334/927 (36)
**Sub-total**			**670**	**1,795**	**1,795/2,465 (72.8)**	**744**	**744/1,795 (41.4)**
High	Kagera	32.8	553	986	986/1,539 (64.1)	576	576/986 (58.4)
	Mtwara	42.3	297	883	883/1,180 (74.8)	324	324/883 (36.7)
**Sub-total**			**850**	**1,869**	**1,869/2,719 (68.7)**	**900**	**900/1,869 (48.2)**

Total			5,676	7,199	7,199/12,875 (55.9)	3,149	3,149/7,199(43.7)

Table S2. Summary of mean COI and proportion of polyclonal infection in each of the study regions stratified by transmission strata

Transmission strata	Region	Mean	Median (IQR)	Minimum-Maximum	Number of samples	Proportion of polyclonal infection (%)
**Very low**	Kilimanjaro	1.5	1 (1-3)	1-5	114	43
	Manyara	1.3	1 (1-2.5)	1-4	222	23.4
	Njombe	1.4	1 (1-3)	1-4	297	37.4
**Low**	Dar Es Salaam	1.4	1 (1-2)	1-3	266	36.5
	Dodoma	1.4	1 (1-2)	1-6	235	23.8
	Songwe	1.5	1 (1-3)	1-4	371	38.8
**Moderate**	Mara	1.5	1 (1-4)	1-5	334	41.6
	Tabora	1.5	1 (1-3)	1-5	410	40.7
**High**	Kagera	2.2	2 (1-15)	1-22	576	61.1
	Mtwara	1.7	1 (1-4)	1-6	324	31.2

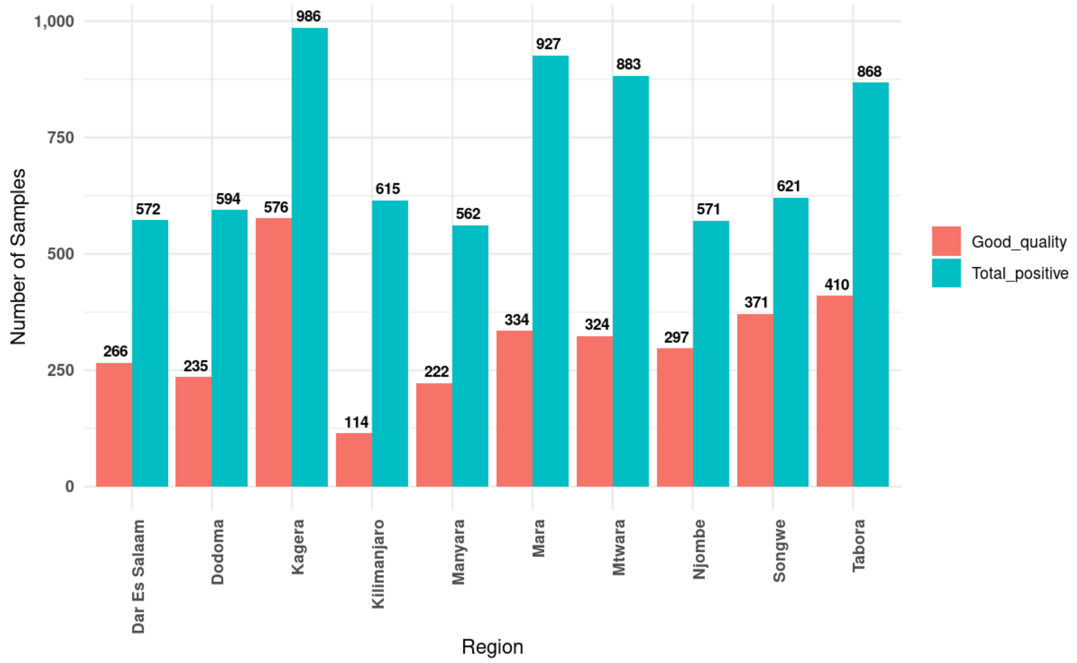

Figure S1: Bar chart showing the regional distribution of parasite samples sequenced (sky blue colour) and good quality samples (salmon colour).

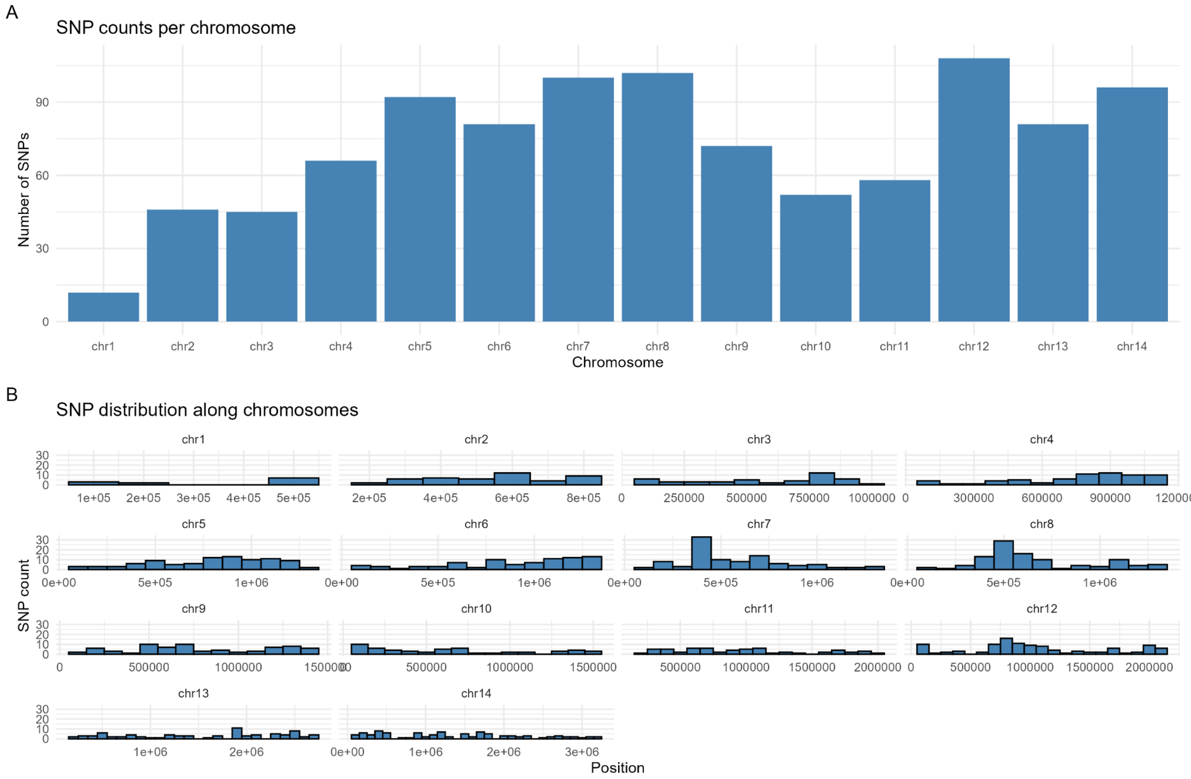

Figure S2: Histogram showing the distribution of SNPs across the 14 chromosomes of *P. falciparum*, with (A) showing the number of retained SNPs per chromosome and (B) showing the SNPs distribution for each chromosome from chromosome 1 to 14.

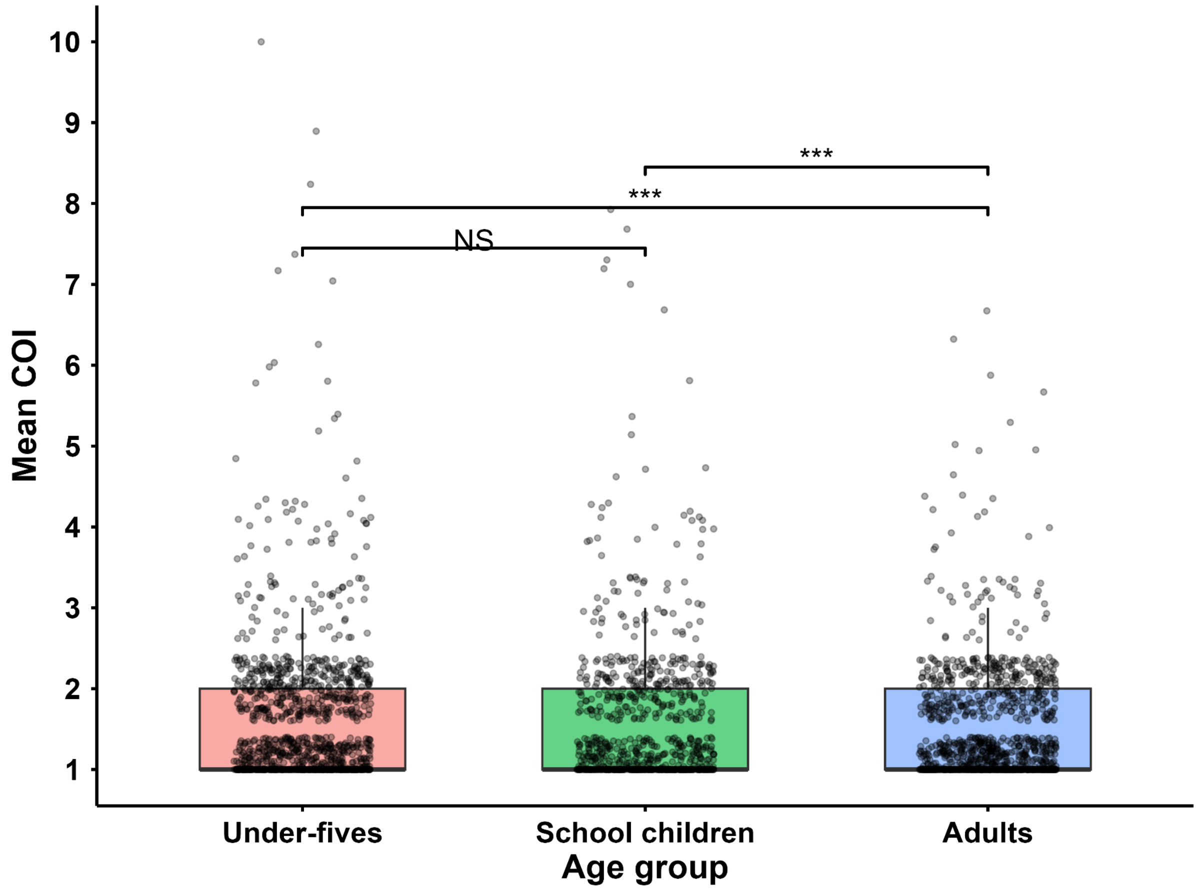

Figure S3: Boxplots showing the distribution of mean COI per age group, with a higher mean COI observed among under-fives and school children compared to adults. The boxplots depict the median (center line), interquartile range (IQR, upper and lower quartiles), whiskers, and outliers (points)

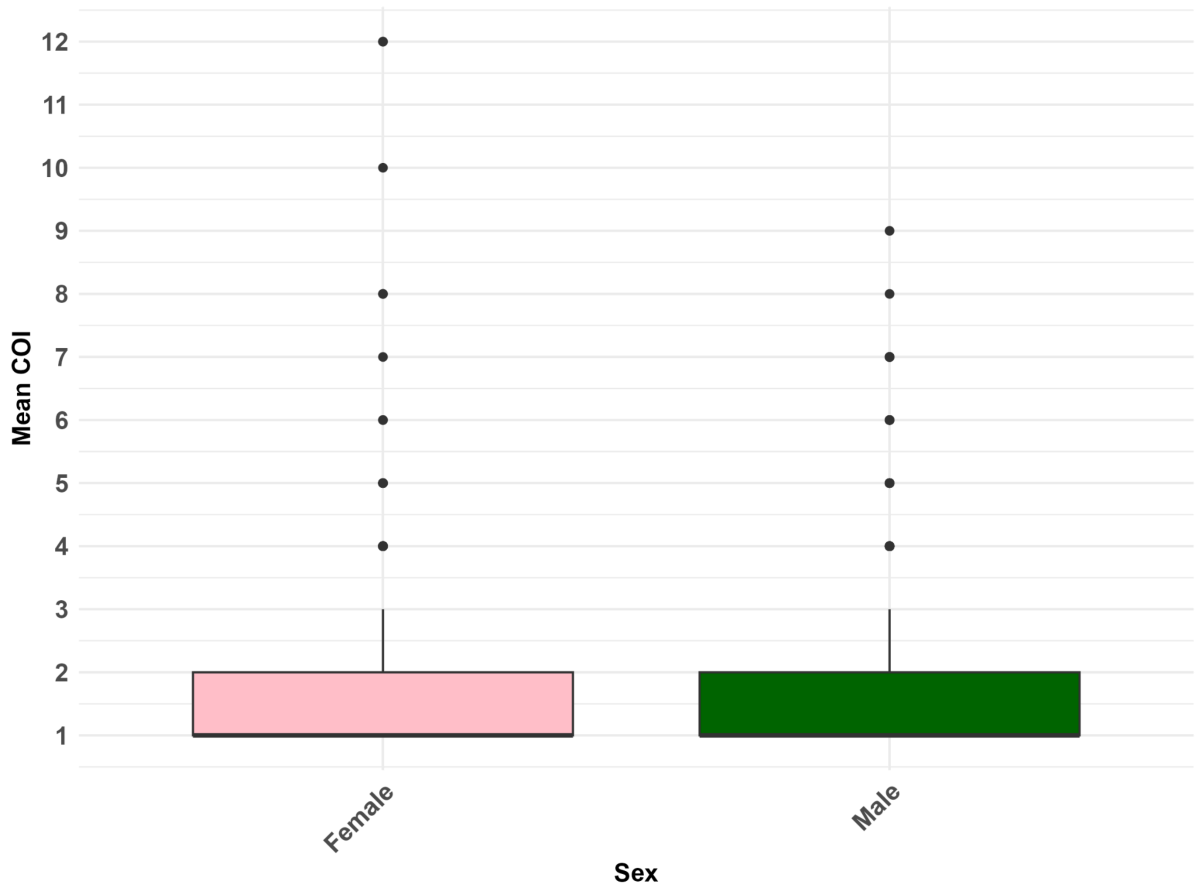

Figure S4: Boxplots showing the distribution of mean COI per sex. Boxplots depict the median (center line), interquartile range (IQR, upper and lower quartiles), whiskers, and outliers (points), with a mean COI ranging from 1 to 13 among female participants and 1 to 9 among male participants.

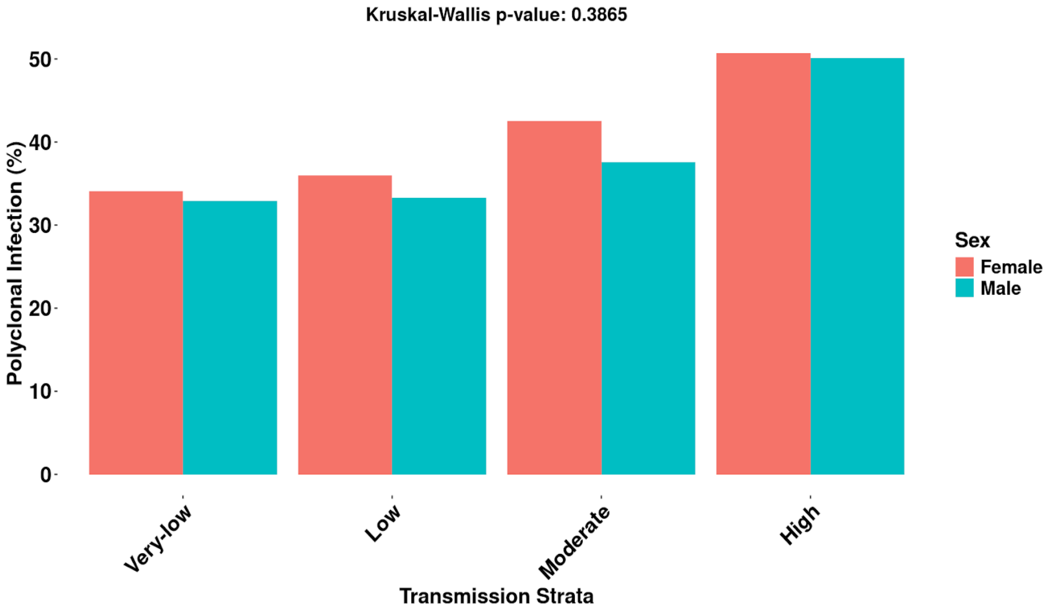

Figure S5: Bar chart showing the proportion of polyclonal infections by sex across different transmission strata. The proportion of polyclonal infections was similar between males and females across all transmission strata. Sky blue colour represents males, and salmon colour represents females.

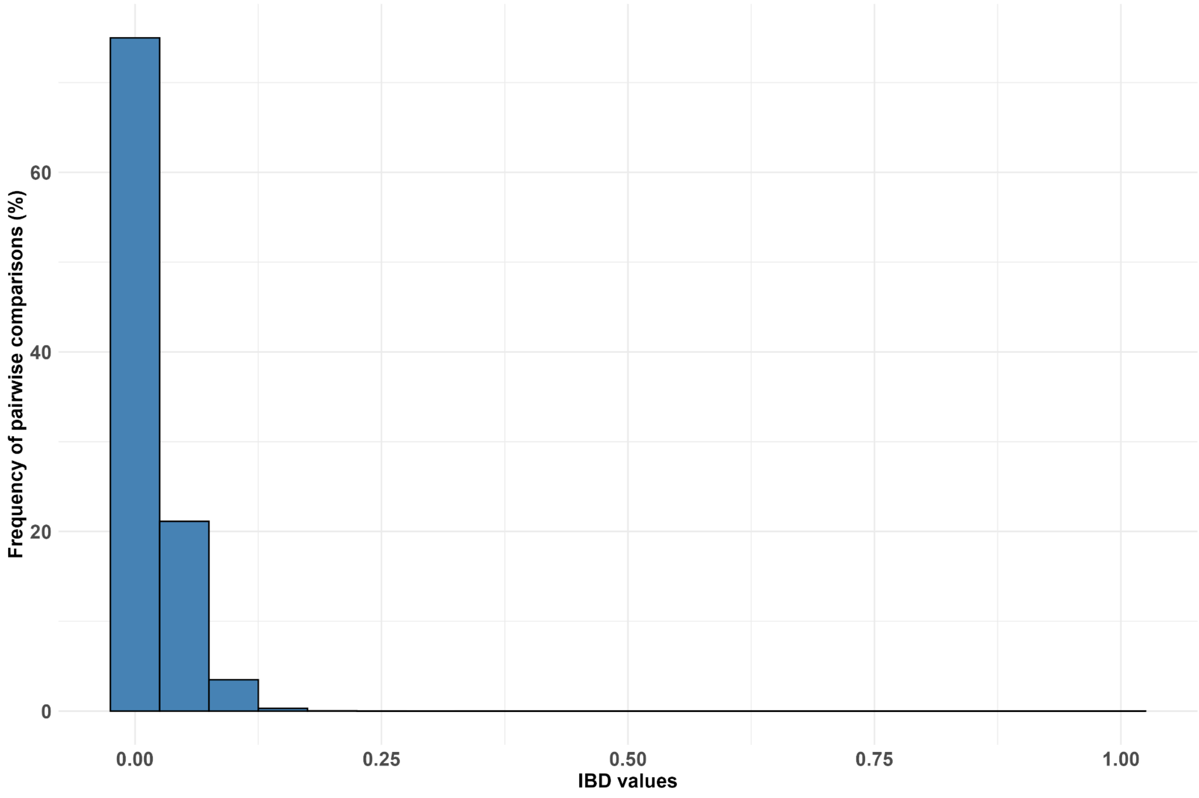

Figure S6: Histogram showing the overall distribution of pairwise IBD values across all parasite sample pairs in the studied regions. The x-axis represents IBD values, and the y-axis represents the frequency of pairwise comparisons.

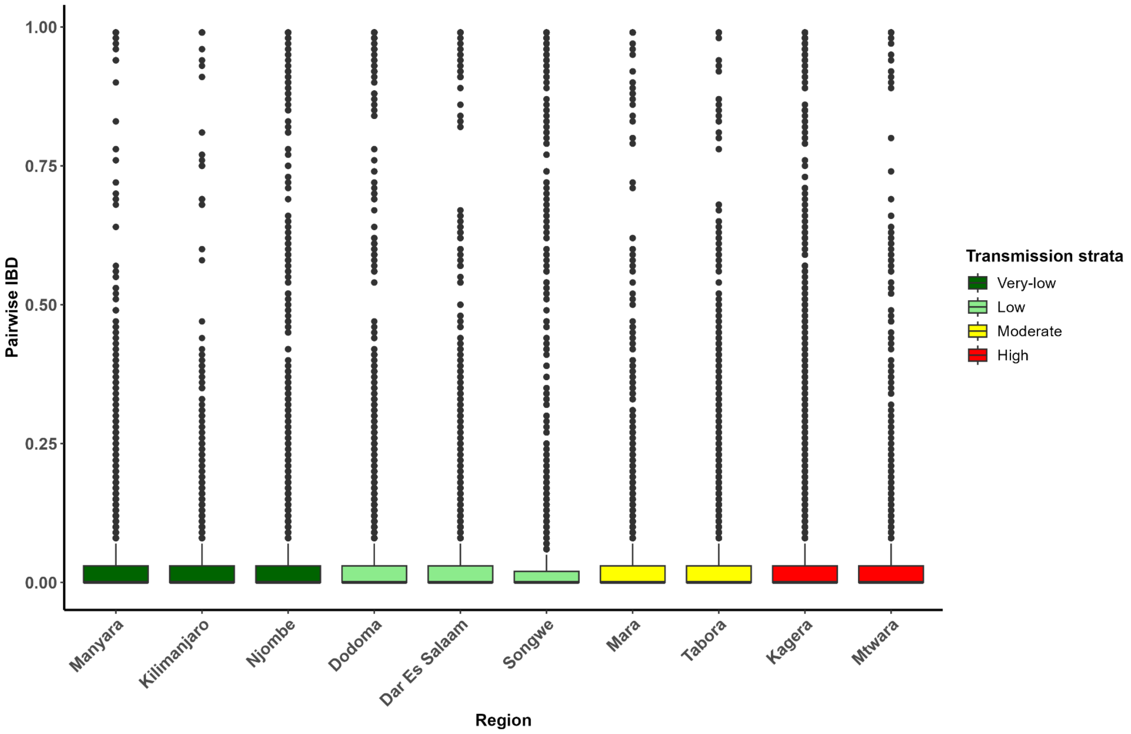

Figure S7: Boxplot showing overall pairwise IBD values among parasites per region across transmission strata. Boxplots depict the median (center line), interquartile range (IQR, upper and lower quartiles), whiskers, and outliers (points). Regions are coloured according to their malaria transmission strata (dark green = very low, green = low, yellow = moderate, and red = high transmission).

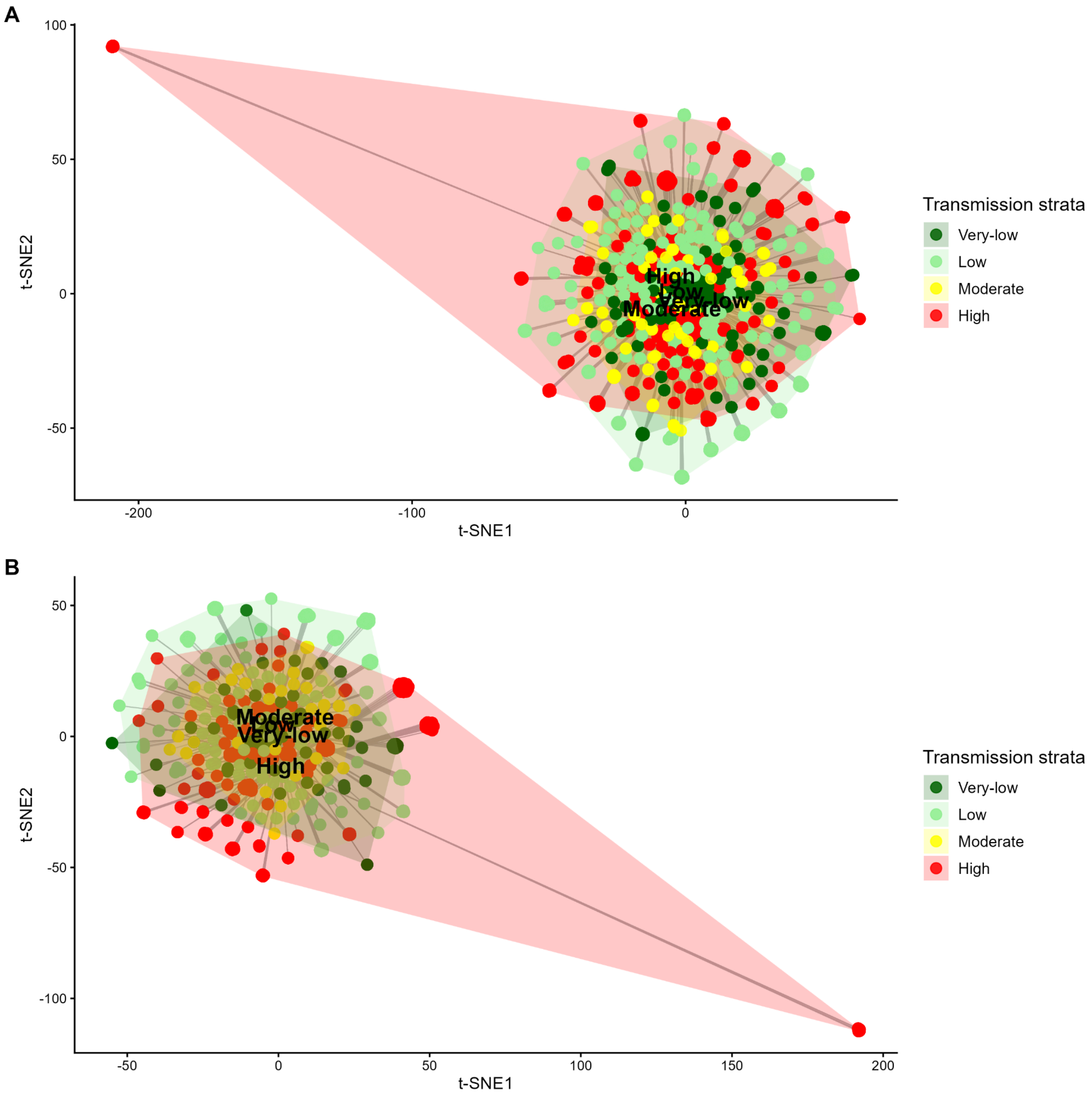

Figure S8: Two-dimensional *t*-SNE visualization of highly related *P. falciparum* parasite samples based on identity-by-descent (IBD). **(A)** Parasite samples sharing IBD ≥ 0.5 and **(B)** parasite samples sharing IBD ≥ 0.9 were projected into two-dimensional space using pairwise genetic distances (1 − IBD). Each point represents an individual parasite sample and is colored by transmission stratum (Very Low, Low, Moderate, High). Shaded convex hulls delineate the distribution of samples within each transmission stratum, while text labels indicate stratum centroids (mean positions). Grey line segments connect individual samples to their respective centroids, illustrating within-stratum clustering and dispersion of highly related parasites. Overlap among strata reflects limited genetic

differentiation across transmission settings.

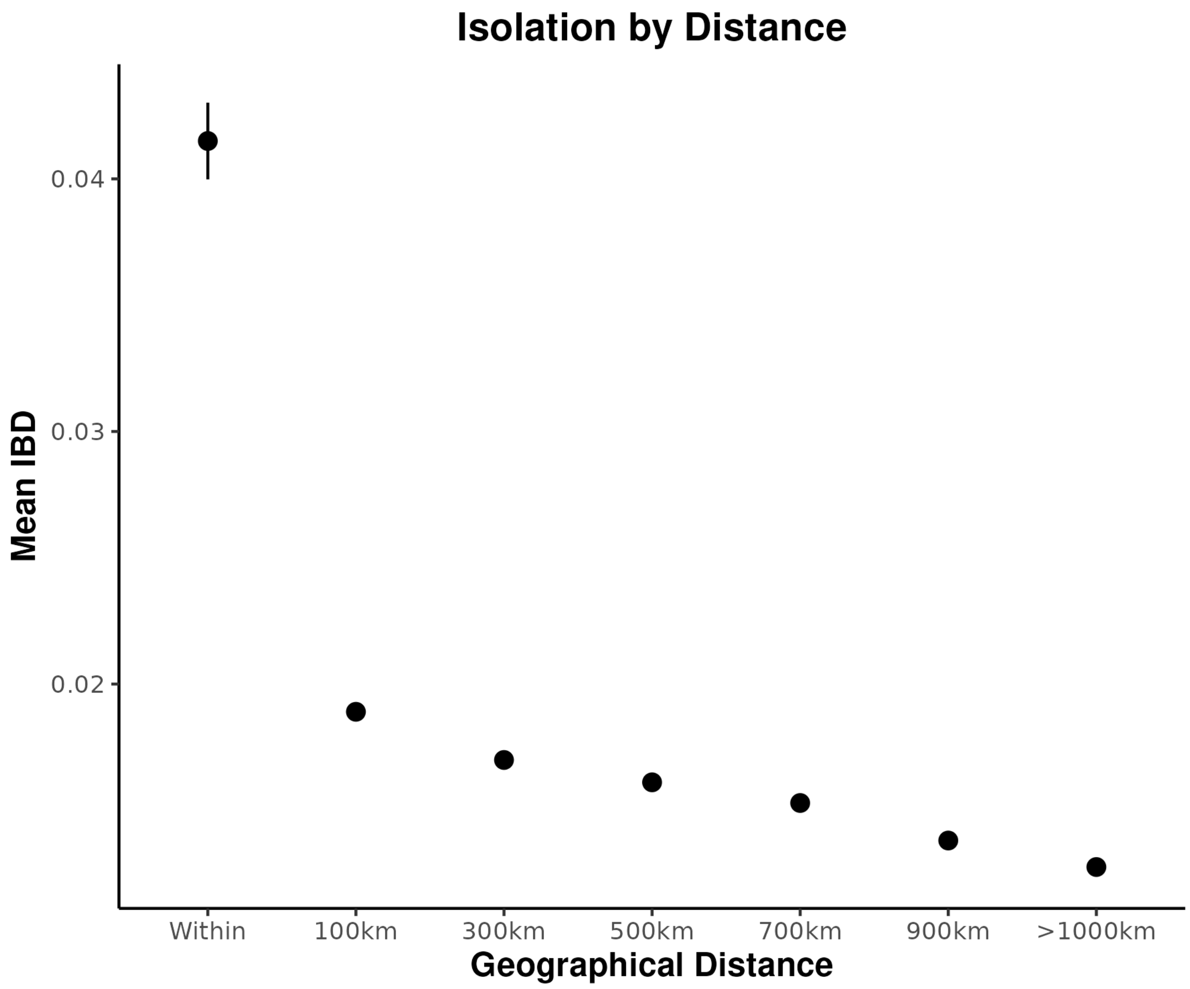

Figure S9: The effect of spatial distance on parasite IBD sharing among regions. IBD-relatedness decreased with increasing geographical distance.

A

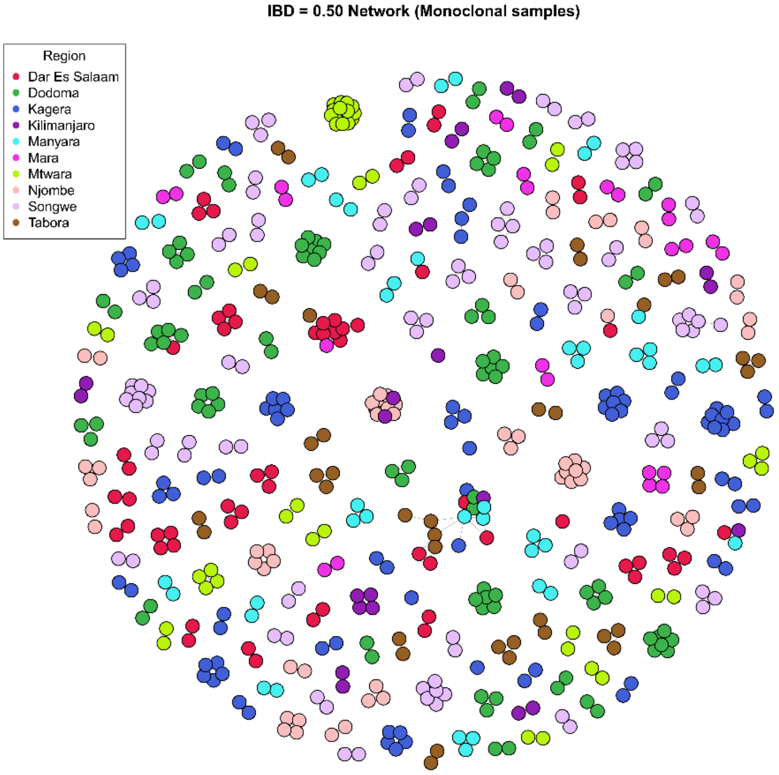
IBD = 0.50 Network (Monoclonal samples)
Region
Dar Es Salaam
Dodoma
Kagera
Kilimanjaro
Manyara
Mara
Mtwara
Njombe
Songwe
Tabora

B

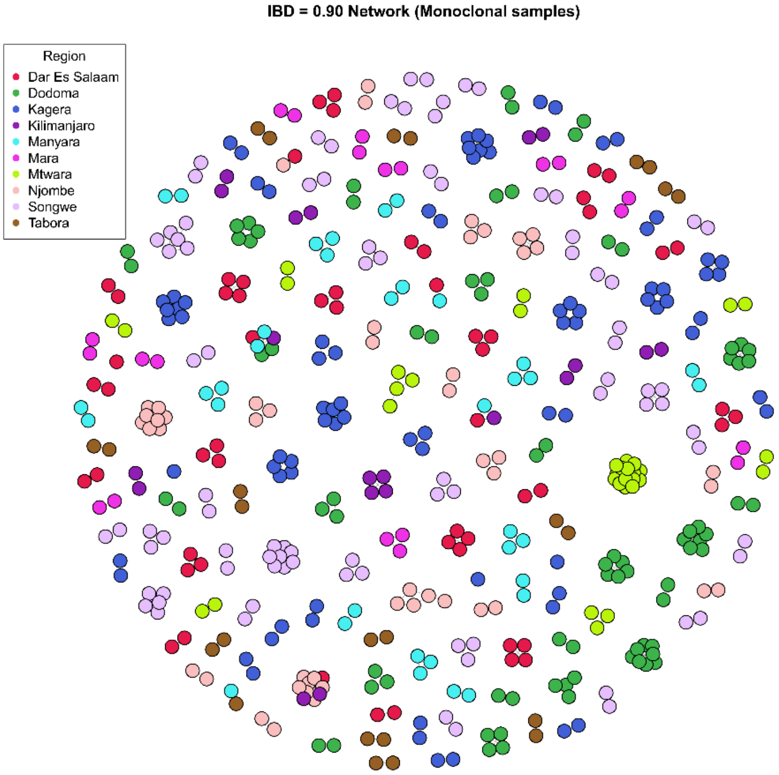
IBD = 0.90 Network (Monoclonal samples)
Region
Dar Es Salaam
Dodoma
Kagera
Kilimanjaro
Manyara
Mara
Mtwara
Njombe
Songwe
Tabora

Figure S10: Identity-by-descent (IBD) network analysis. Each node represents an individual monoclonal parasite isolate (COI = 1), and edges connect parasite pairs exceeding the specified pairwise IBD threshold. Node colours indicate the region of origin. **(A)** IBD ≥ 0.50: The network shows multiple connected components with moderate clustering, primarily structured within regions, with limited inter-regional links. **(B)** IBD ≥ 0.90: The network is highly fragmented, consisting of small, disconnected clusters with no large connected component, indicating localized transmission and limited long-distance parasite movement.

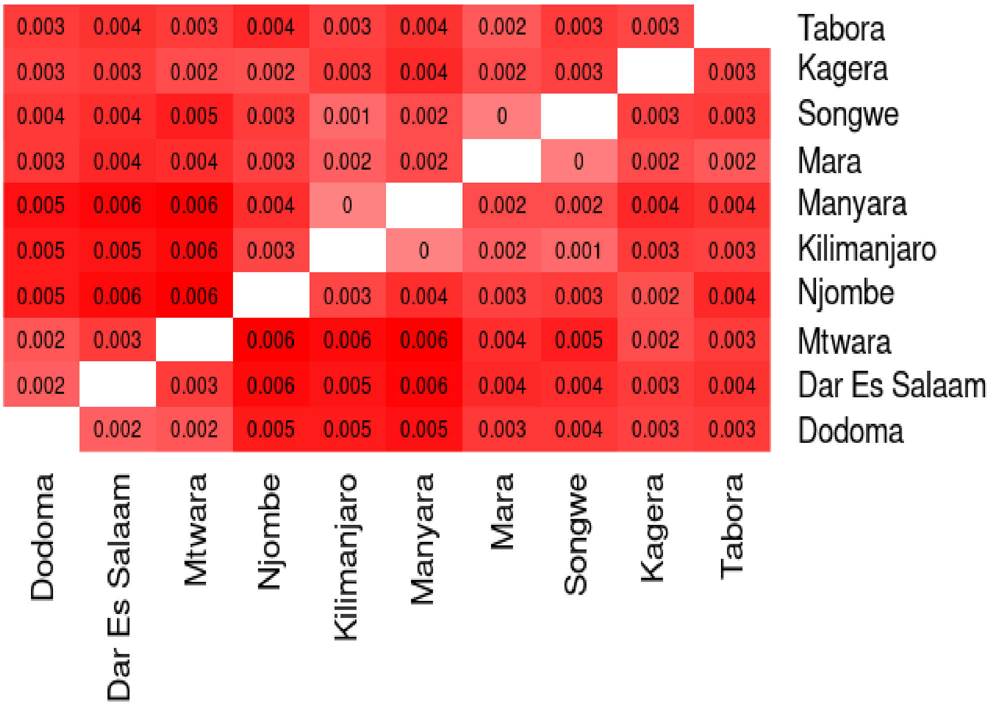

Figure S11: The heatmap showing parasite genetic differentiation among regions using pairwise $F_{ST,}$ with F_{ST} values ranging from 0 to 0.006 between regions

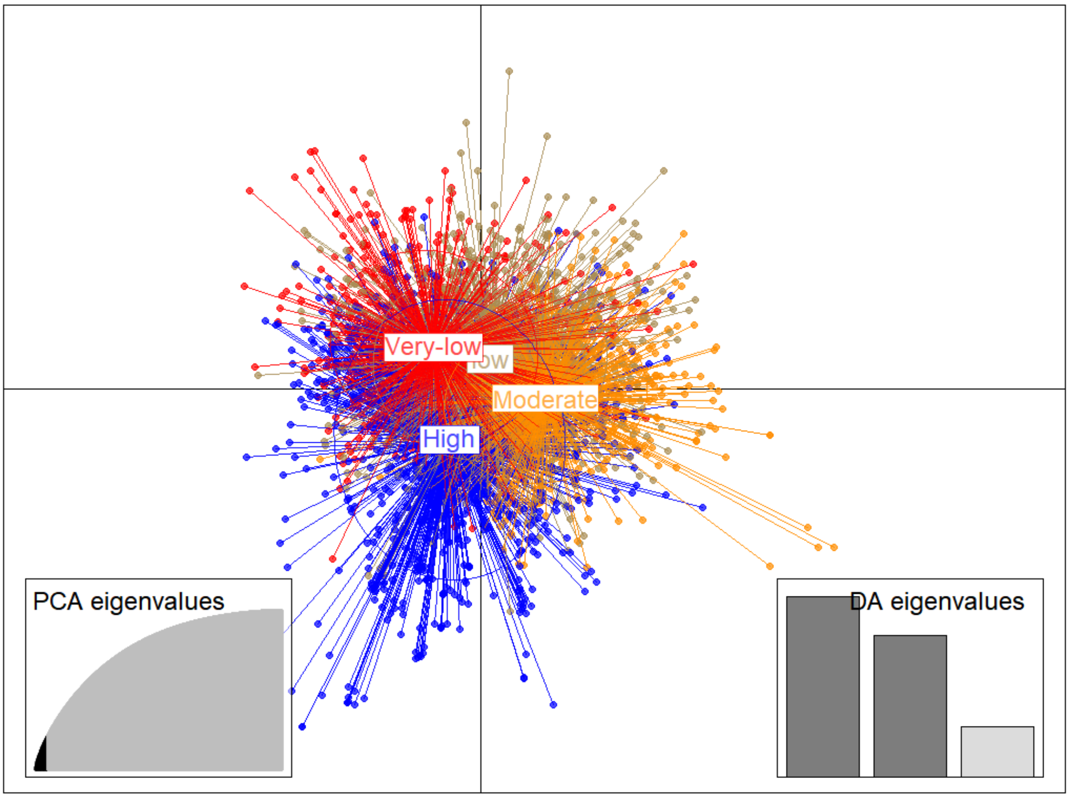

Figure S12: DAPC plot showing parasite population structure across transmission strata

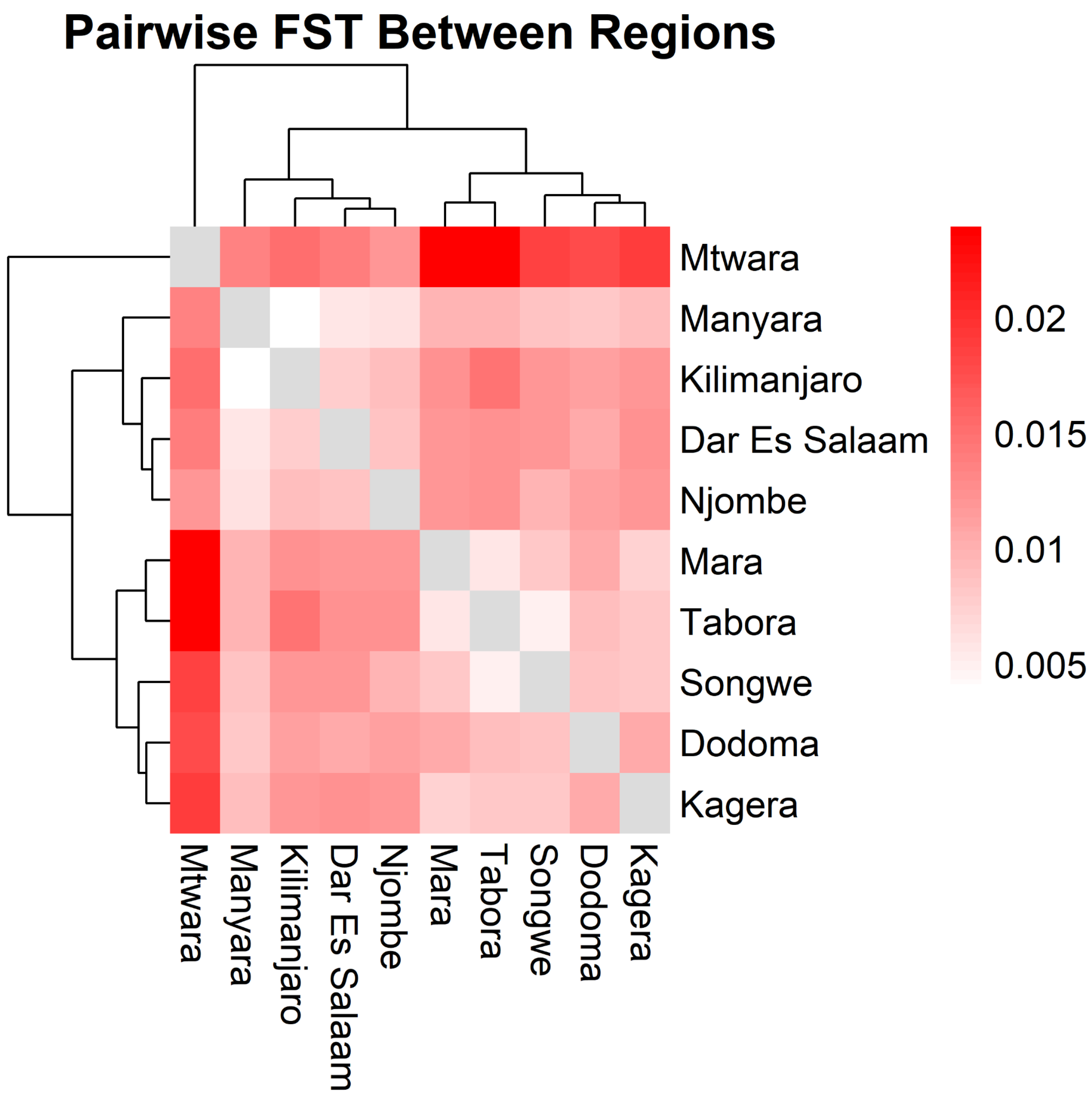

Figure S13: Pairwise F_{ST} heatmap among regions using monoclonal samples. Pairwise F_{ST} values, indicating genetic differentiation, are shown between regions. Most regions exhibit very low F_{ST} (~0.005–0.01), reflecting high genetic similarity.

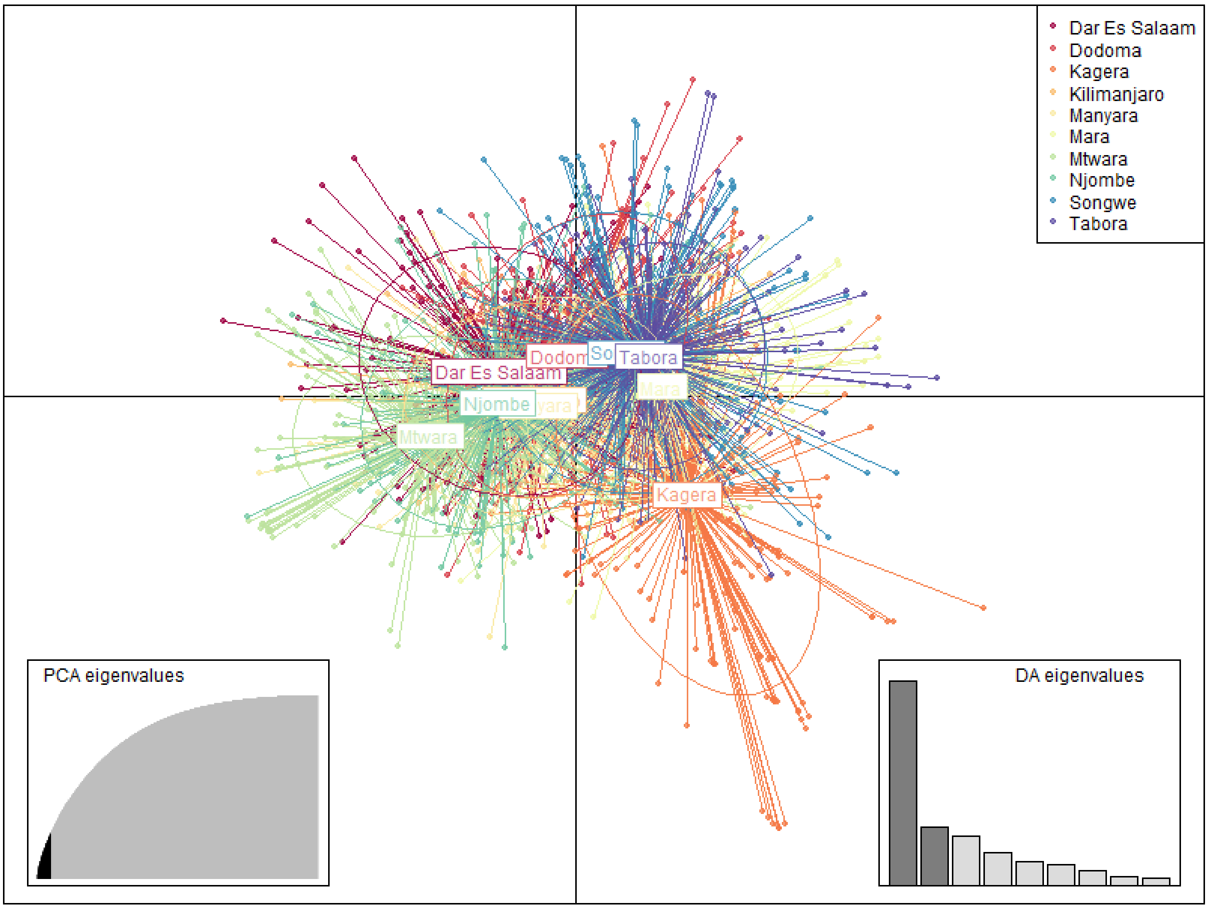

Figure S14: DAPC plot showing parasite population structure across regions using monoclonal samples for the sensitivity test.
